# ZNF276 promotes the malignant phenotype of breast carcinoma by activating the CYP1B1-mediated Wnt/β-catenin pathway

**DOI:** 10.1038/s41419-022-05223-8

**Published:** 2022-09-10

**Authors:** Ting Lei, Wenwu Zhang, Yongyin He, Shi Wei, Xiaoyu Song, Yi Zhu, Guoqing Luo, Zhenzhan Kuang, Guanjie Li, Quan Zhou, Zhaohui Sun, Bin Xiao, Linhai Li

**Affiliations:** 1 Department of Laboratory Medicine, The Sixth Affiliated Hospital of Guangzhou Medical University, Qingyuan People’s Hospital, 511518 Qingyuan, China; 2 Department of Clinical Laboratory, The Fifth Affiliated Hospital of Sun Yat-sen University, 519000 Zhuhai, Guangdong China; 3 Guangzhou University of Chinese Medicine, 510405 Guangzhou, China; 4 Department of Histology and Embryology, School of Basic Medical Sciences, Guangzhou Medical University, Guangzhou, Guangdong China; 5 Department of Laboratory Medicine, General Hospital of Southern Theatre Command of PLA, 510010 Guangzhou, China; 6 Department of General Surgery Section 5, The Sixth Affiliated Hospital of Guangzhou Medical University, Qingyuan People’s Hospital, 511518 Qingyuan, China

**Keywords:** Breast cancer, Transcriptional regulatory elements

## Abstract

Zinc finger proteins (ZNFs) have been demonstrated to participate extensively in breast cancer progression by functioning as transcription factors, but there are still a variety of ZNFs whose biological mechanisms remain unknown. Here, we show that zinc finger protein 276 (ZNF276) is highly expressed in breast cancer tissues and cell lines. Higher level of ZNF276 correlated with poor prognosis. Gain-of and loss-of function suggested that ZNF276 is essential for the proliferation, migration and invasion of breast cancer cells in vitro and metastasis in vivo. RNA-sequencing and CUT&Tag assay revealed that ZNF276 controlled a variety of growth and metastasis-related genes expression. ZNF276 transcriptionally promoted the expression of CYP1B1 by directly binds to the promoter region of the CYP1B1 through its C_2_H_2_ domain. ZNF276 facilitated the translocation of β-catenin from cytoplasm to nucleus through CYP1B1, leading to the upregulation of cyclin D1 and c-Myc, and the activation of the Wnt/β-catenin pathway. Knockdown of CYP1B1 significantly blocked the ZNF276-mediated effects on cell proliferation, migration and invasion. Lastly, ZNF276 interacted with MAGEB2 which enhanced the binding of ZNF276 at the CYP1B1 promoter, promoted CYP1B1 expression and Wnt signaling activation. Collectively, these findings highlight the oncogenic role of ZNF276 on breast cancer cell proliferation and metastasis. Targeting ZNF276/MAGEB2 axis may serve as a potential therapeutic strategy for breast cancer patients.

## Introduction

Breast cancer has replaced lung cancer as the most common malignancy since 2020, according to the cancer burden statistical data reported by the International Agency for Research on Cancer [1]. Metastasis is still the leading cause of breast cancer-related death. It is difficult for multiple metastasis-driven genes to become drug targets because we have revealed only part of the entire complex metastasis mechanism. Novel targets and the underlying mechanisms still need to be explored.

Zinc finger proteins (ZNFs) are the largest transcription factor family in eukaryotic cells. Most members of this family are aberrantly expressed in cancer. Many ZNFs are involved in key processes in breast cancer cells, such as proliferation, apoptosis, metastasis, drug resistance, autophagy and stem cell property promotion [2–4]. ZNF276 is a member of ZNF family whose biological effect in various malignant tumors is largely unknown. The coding gene of ZNF276 lies in chromosome 16q23.4 where loss of heterozygosity frequently occurs in breast tumors [5]. Mutation analysis of its coding region suggested that ZNF276 might be a tumor suppressor, however, there is no as of yet definitive evidence on its role in breast cancer.

Cytochrome P450 1B1 (CYP1B1) is a monooxygenase that is highly expressed in many tumors, such as brain, breast and colon tumors [6], which has led to a strong interest in the role of CYP1B1 in tumorigenesis. It has been shown that overexpression of CYP1B1 is associated with increased cancer risk through pro-inflammatory cytokines, metastasis, and disturbances in the regulation of cell proliferation, migration and differentiation [7, 8]. In addition, overexpression of CYP1B1 has been associated with increased tumor size, increased tumor grade, frequent lymph node metastases and lymphovascular invasion [9]. Although CYP1B1 expression is significantly correlated with the activation of Wnt/β-catenin pathway [10], the transcriptional regulation mechanism of CYP1B1 remains unclear.

Melanoma-Associated Antigen B2 (MAGEB2) belongs to the melanoma antigen gene (MAGE-I) family of tumor-specific antigens and is associated with several tumor diseases, such as head and neck squamous cell carcinoma, laryngeal cancer and nerve sheath tumors [11–13]. MAGEB2 could bind to the NH2-terminal NTD domain of AR through the F-box in the MAGE homology domain, and promotes prostate cancer cell growth by upregulating its downstream targets PSA and NX3.1 to activate AR signaling [14]. MAGEB2 has been shown to promote the transcription of ribosomal rRNA and transcription factor E2F, but the mechanism of how MAGEB2 forms a transcriptional complex to cooperatively regulate the expression of downstream genes remains unknown.

In this study, we found that the protein expression of ZNF276 was upregulated in breast cancer tissues and cells. Overexpression of ZNF276 promoted, while silencing of ZNF276 inhibited, the proliferation, migration and invasion of breast cancer cells. ZNF276 recruits MAGEB2 to facilitate the transcription of CYP1B1 and the activation of the Wnt/β-catenin pathway, leading to a malignant breast cancer phenotype. Our study shed light on the crucial role and mechanism of ZNF276 in the progression of breast cancer in mediating cell proliferation and metastasis.

## Materials and methods

### Cell lines and cell culture

All cell lines, including HEK293T, MCF-10A, MCF-7, MDA-MB-231, SK-BR-3, BT-474, T-47D, HS578T, and UACC-812 cells, were obtained from the cell bank of the Shanghai Institute of Biological Sciences and the Chinese Academy of Sciences. HEK293T, MCF-7, MDA-MB-231, SK-BR-3, T-47D, HS578T, and UACC-812 cells were grown in Dulbecco’s modified Eagle’s medium (DMEM) with 10% foetal bovine serum (FBS) (Gibco, Detroit, MI, USA). BT-474 cells were cultured in RPMI 1640 (Gibco) with 10% FBS, and MCF-10A cells were cultured in mammary epithelial cell basal medium (MEBM) (Lonza, Switzerland). Cells were maintained at 37 °C in a humidified atmosphere of 5% CO_2_.

### Construction of ZNF276 stably overexpression and knockout cells

ZNF276 stably overexpressing cell line was generated by lentivirus infection in SK-BR-3 and MDA-MB-231. Lentivirus was produced by co-transfection the expression plasmid (Flag-pCDH-ZNF276) and the packing plasmids (pMD2.G and psPAX2) into HEK293T cells. The supernatants were collected after 48 h and filtered through 0.45 μm filters (Millipore). The virus was applied to infect SK-BR-3 and MDA-MB-231, and stable transfection cell lines were selected with 2 μg/ml puromycin for 15 days.

UACC-812 cells were used to construct stably ZNF276 knockout cells. The single guide RNA (sgRNA) sequence targeting ZNF276 gene were designed by the online program CRISPOR (http://crispor.tefor.net/). The sgRNA (Sequence: 5′-TGGGTCGTCCCGACAGTGCG-3′) was inserted into the Px0001 vector. The Px0001-sgRNA-ZNF276 and Px0002 were co-transfected into UACC812 cells using Lipofectamine 3000 (Invitrogen, Carlsbad, CA, USA). Positive transfected monoclonal cells were selected using 2 µg/mL puromycin. The efficiency of ZNF276 knockout was verified by gene sequencing and western blot.

### Cell transfection

Flag-pCDH empty vector, Flag-pCDH-ZNF276, KO-NC, KO-ZNF276, HA-MAGEB2, HA-CYP1B1, pGL3-CYP1B1 promoter wildtype and region 1–9 deletion, ZNF276 region 1–6 deletion, pGL3-Basic, pRL-TK, and TOP/FOP Flash plasmids were constructed by Focus Biology (Nanchang, China). Control siRNA, siRNA-ZNF276 1–3 and siRNA-CYP1B1 1–3 were purchased from RiboBio (Guangzhou, China). Control sgRNA and sgRNA-ZNF276 were obtained from Genescript (Nanjing, China). Lipofectamine 3000 was used for plasmid and siRNA transfection according to the manufacturer’s instructions. Cas9 protein was an important component of sgRNA transfection in the CRISPR RNP liposome transfection method.

### Tissue samples

A total of four groups of tissue samples were applied in this study. Tumor tissues and matched surrounding normal tissues from 10 independent breast cancer patients (female, 27–51 years old) were used for validating ZNF276 mRNA and protein expression by quantitative real-time polymerase chain reaction (RT-qPCR) and western blot. The clinical characteristics of these samples were presented in Table S1. Three tissue microarrays were purchased from Shanghai Otto Biotechnology Co. Tissue microarray containing 118 breast cancer tissues and 48 adjacent tissues was used to analyze the expression of ZNF276 by immunohistochemistry. An additional chip containing 74 paraffin-embedded samples and 6 adjacent tissues was used to quantify CYP1B1 expression level. Tissue microarray containing 132 breast cancer patients with 10 years follow-up and prognostic information was used for prognostic analysis.

Written informed consent was obtained from each patient. These human studies were performed in accordance with the Declaration of Helsinki and were approved by the Ethics Committee of Qingyuan People’s Hospital (IRB-2022-010).

### Immunohistochemistry

The breast cancer tissues were fixed with 10% formalin and embedded in paraffin before the sections were treated with specific primary antibodies. After antigen repair, the sections were blocked with 1% BSA (Thermo Fisher, CA, USA) in PBST buffer at room temperature for 2 h, then incubated at 4 °C overnight with the primary antibody. The sections were washed three times and incubated with HRP-polymer-conjugated secondary antibody (Thermo Fisher, CA, USA) at room temperature. Then the sections were stained with 3,3-diaminodbenzidine substrate and hematoxylin. The primary tumors from the breast fat pads of nude mice were fixed in 4% paraformaldehyde and then embedded in paraffin. The tissue sections were incubated with anti-ZNF276 (1:1000, NBP1-82681, Novusbio, USA) antibody. The staining intensity was evaluated by H-score (H-score = ∑[PI (percentage of positive cells) × I (intensity)] = percentage of cells of weak intensity × 1 + percentage of cells of moderate intensity × 2 + percentage of cells of strong intensity × 3). According to the mean value of H-score, breast cancer samples were divided into high and low ZNF276 expression groups to analyze the correlation between ZNF276 and clinical features, as well as the prognosis of breast cancer patients.

### RNA isolation and quantitative real-time PCR

Total RNA was extracted from tissue samples or cells using TRIzol (Takara, Kusatsu, Japan). After measuring the RNA concentration, cDNA was synthesized using a reverse transcription kit (Takara). A SYBR Green Master Mix Kit (Accurate Biotechnology Co., Ltd) was utilized to perform RT-qPCR in CFX96^TM^ (Bio-Rad Laboratories, Inc.). All mRNA expression levels were defined according to the threshold cycle (Ct), and relative expression levels were calculated using the 2^−△△Ct^ method after normalization to GAPDH expression. The primer sequences are listed in Table S2.

### Immunoblotting and immunoprecipitation

GAPDH was used as an internal control. We used the following primary antibodies: anti-ZNF276 (1:1000, PA5-54578, Invitrogen), anti-CYP1B1 (1:1000, ab33586, Abcam), anti-β-catenin (1:1000, #8480, CST), anti-cyclin D1 (1:1000, #2978, CST), anti-cMyc (1:1000, #5605, CST), anti-HA (1:1000, #3724, CST), anti-Flag (1:1000, #14793, CST) and anti-GAPDH (1:1000, #5174, CST). Anti-mouse IgG (1:1000, #7076, CST), anti-rabbit IgG (1:1000, #7074, CST) and guinea pig anti-rabbit IgG (H&L) antibodies (1:100, #101961, ABIN, Beijing, China) were used as the secondary antibodies. Expression was quantified using densitometry and ImageJ software.

Co-IP assays were performed according to the manufacturer’s protocol (Beaver, Suzhou, China). Briefly, total cell extracts were precleared and incubated with anti-Flag or anti-HA with gentle shaking for 15 min at 4 °C, followed by the addition of protein A/G beads overnight. The suspensions were washed and resuspended in 1× loading buffer, boiled for 5 min and magnetically separated. The supernatants were collected for SDS-PAGE.

### Cell counting kit-8 (CCK-8), transwell, wound healing, and clonogenic assays

For the CCK-8 assay, cells were seeded at a density of 5000 cells per well in a 96-well plate, and cell viability was measured using CCK-8 (Beyotime, Shanghai, China) at a certain time point after transfection.

Transwell invasion assays were performed using a 24-well chamber with Matrigel (Corning, USA), whereas migration assays were performed without Matrigel. Cells were incubated in the upper chamber with serum-free medium for 48 h. Medium supplemented with 10% FBS was added to the lower chamber as the chemoattractant. Cells that penetrated into the lower chamber were photographed under a microscope and counted.

For the wound healing assay, wounds were created in confluent areas in cultures with a cell density below 90%. Wound healing within the scrape line was observed at a certain time point, and representative scrape lines for each cell line were photographed. The optical wound distances were measured using ImageJ software.

For the clone formation assay, cells were seeded in a 6-well plate (1000 per dish) and incubated for 14 days. Colonies were washed with phosphate buffered saline (PBS), fixed with 4% paraformaldehyde, and stained with 0.5% crystal violet before counting the number of colonies consisting of >50 cells. All experiments were performed in triplicate.

### Orthotopic breast cancer and lung metastasis mouse model

MDA-MB-231 cells (1 × 10^6^ cells in 0.1 ml PBS) were injected subcutaneously into 4-week-old female Balb/c nude mice. Mice were randomized into experimental and control groups, with 6 mice in each group. The tumor diameter in the nude mice was measured every 5 days. After 30 days, all mice were sacrificed, and the tumor weights and sizes were measured. The volume of the tumor was calculated by the following formula: volume = (width^2^ × length)/2. Immunohistochemistry was subsequently performed to analyze the expression of Ki67 and ZNF276 in tumor tissues. All nude mice were purchased from the Guangdong Medical Laboratory Animal Center, and the Guangdong Provincial Laboratory Animal Quality Certificate was obtained. For the metastasis analysis, 3 × 10^6^ cells were injected into the tail vein of 6 weeks old female BALB/c nude mice (6 mice per group). The number of surface metastatic foci in lungs were counted. In vivo bioluminescent imaging was conducted to monitor lung metastases.

### Confocal microscopy

Cells were fixed with 4% paraformaldehyde, blocked with 5% BSA, and incubated with anti-Flag (1:1600, #8146, CST) and anti-β-catenin antibodies (1:100, #8480, CST) overnight at 4 °C, followed by incubation with anti-rabbit IgG (Alexa Fluor 488 Conjugate) (1:1000, #4412, CST) and anti-mouse IgG (Alexa Fluor 594 Conjugate) (1:1000, #8890, CST) at room temperature for 1 h. Cells were counterstained with 4’,6-diamidino-2-phenylindole (DAPI). Confocal microscopy was performed using a Radiance 2000 laser scanning confocal microscope (Carl Zeiss, Germany).

### RNA sequencing and cleavage under targets and tagmentation (CUT&Tag)

RNA sequencing was carried out to detect the potential target genes of ZNF276. Total RNA was extracted from MDA-MB-231 cells stably expressing ZNF276 or empty vector. Quality control of cDNA libraries was performed by a bioanalyzer instrument after construction. Strand-specific RNA sequencing was performed on a HiSeq 2000 instrument (Illumina). Differential expression analysis, such as clustering, functional annotation and functional enrichment, were performed based on gene expression in the two sample groups, with fold change (FC) ≥ 2 and false discovery rate (FDR) < 0.05 as screening criteria.

CUT&Tag was performed in the standard manner to explore the binding DNA of ZNF276. Specific DNA fragments were obtained from MDA-MB-231 cells overexpressing ZNF276 and purified using a NovoNGS^®^ CUT&Tag High-Sensitivity Kit (Novoprotein Scientific Inc.) following the manufacturer’s recommended protocol. The resulting DNA libraries were quantified and sequenced on the Illumina HiSeq 2000 platform, and downstream processing of sequencing data was analyzed by bioinformatics analysis. ChIP-PCR was used to confirm the binding of ZNF276 to the promoter of the target gene. Related primers are listed in Table S2.

### Dual-luciferase assay

A pGL3 reporter containing target regions was transfected into breast cancer cells using a Renilla luciferase vector. Firefly and Renilla luciferase activities were detected using a Dual-Luciferase Reporter Assays Kit (#E1910, Promega, Madison, WI, USA) at 48 h post-transfection according to the manufacturer’s instructions.

The TOP/FOP Flash assay was performed in the same manner. TOP Flash contains three copies of the TCF-4 binding sites, and FOP Flash (the control reporter) contains mutated TCF-4 binding sites. The Renilla luciferase expression plasmid acted as the system control.

### Statistical analysis

Statistical analysis was performed using SPSS 20.0 and GraphPad Prism 7.0. The data are presented as the mean ± SD of three independent experiments. Student’s *t* test was used for studies with two-group comparisons. Univariate and multivariate analyses were performed using Cox’s proportional hazard model to evaluate the correlation between ZNF276 expression and patient prognosis. *P* < 0.05 was considered statistically significant. * indicates *P* < 0.05, ** indicates *P* < 0.01 and *** indicates *P* < 0.001 in all figures.

## Results

### ZNF276 is highly expressed in breast cancer tissues and cell lines

**Fig. 1:**
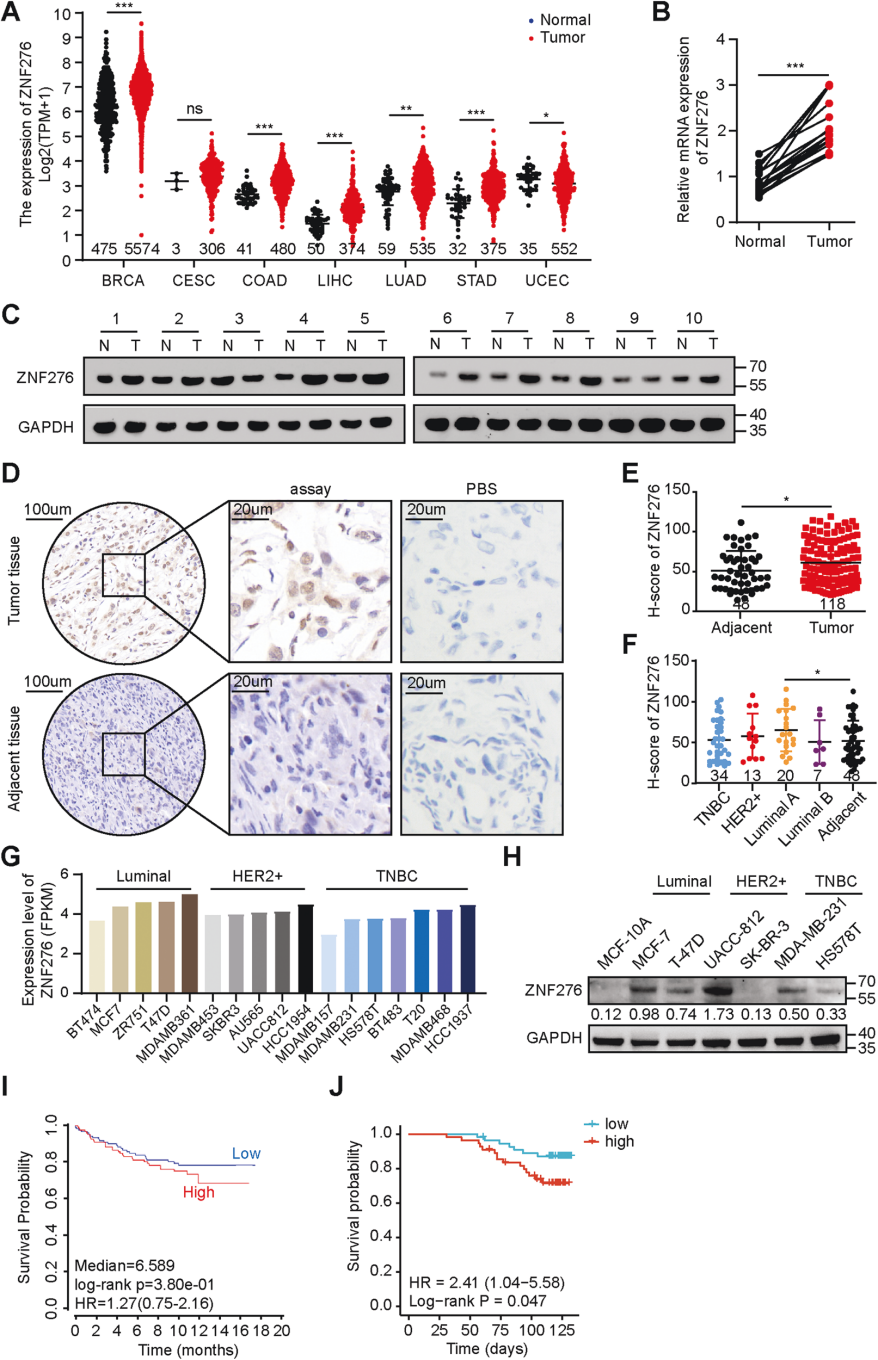
Expression and prognosis of ZNF276 in breast cancer tissues and cell lines. **A** mRNA expression of ZNF276 in different malignancies and normal tissues from TCGA database. BRCA breast carcinoma. CESC Cervical and endocervical carcinoma. COAD Colon adenocarcinoma. LIHC Liver hepatocellular carcinoma. LUAD Lung adenocarcinoma. STAD Stomach adenocarcinoma. UCEC Uterine Corpus Endometrial Carcinoma. **B**, **C** RT-qPCR (**B**) and western blot (**C**) analysis of ZNF276 expression in 10 breast cancer tissues and matched surrounding normal tissues. **D** Representative pictures of ZNF276 expression in 118 breast cancer tissues and 48 adjacent tissues from tissue chips. **E**, **F** Statistical analysis of ZNF276 protein expression in breast cancer tissues and adjacent tissues (**E**) and different molecular subtypes (**F**) from tissue chips by H-score. **G** ZNF276 mRNA expressions in different breast cancer cell lines were obtained from the CCLE portal. **H** Protein expression of ZNF276 was detected in normal breast cells and six breast cancer cells by western blot. **I** Correlation between ZNF276 expression and prognosis of breast cancer patients from TCGA dataset. **J** Kaplan–Meier curve showing the overall survival of 132 breast cancer patients separated by high and low ZNF276 expression. *p < 0.05; **p < 0.01; ***p < 0.001.

To explore the potential role of ZNF276 in breast neoplasia, we performed a comprehensive analysis of ZNF276 expression at the tissue and cellular levels. Firstly, the RNA-seq data from the TCGA database showed that ZNF276 mRNA levels were significantly upregulated not only in breast tumors but also in colon, liver and gastric cancers compared to normal tissues (Fig. 1A). We examined the expression of ZNF276 in 10 breast cancer tissues and paired normal tissues. The results showed that the mRNA and protein levels of ZNF276 were higher in breast cancer tissues than normal tissues (Fig. 1B, C). We further performed an immunohistochemistry analysis using tissue chips containing 118 breast cancer cases and 48 adjacent tumor cases. The immunohistochemical score (H-score) showed that ZNF276 expression was significantly higher in breast cancer tissues than in adjacent tissues (Fig. 1D, E). The expression of ZNF276 was higher in Luminal A and HER2 breast cancer than that in other subtypes (Fig. 1F). In addition, the expression of ZNF276 was positively associated with several clinical characteristics, such as age, WHO grade, TNM grade and clinical stage (Table 1).

**Table 1:** Association between ZNF276 expression and clinicopathologic features of patients with breast cancer (*n* = 118).

Characteristics	ZNF276 expression		Total	*P* value
	Low (*n* %)	High (*n* %)		
Age (years)				0.032*
≤60	45 (57.7%)	33 (42.3%)	78	
60	14 (35%)	26 (65%)	40	
WHO grade				0.046*
I (I–II)	0	4 (100%)	4	
II	32 (46.4%)	37 (53.6%)	69	
III (II–III)	27 (60%)	18 (40%)	45	
Tumor size (cm)				0.579
≤3	34 (52.3%)	31 (47.7%)	65	
>3	25 (47.2%)	28 (52.8%)	53	
TNM grade				
* T*				0.030*
T1	13 (81.3%)	3 (18.7%)	16	
T2	36 (42.4%)	49 (57.6%)	85	
T3	8 (61.5%)	5 (38.5%)	13	
T4	2 (50%)	2 (50%)	4	
* N*				0.370
N0	19 (43.2%)	25 (56.8%)	44	
N1 (pN1)	20 (51.3%)	19 (48.7%)	39	
N2 (pN2)	9 (47.4%)	10 (52.6%)	19	
N3 (pN3)	11 (68.8%)	5 (31.2%)	16	
Clinical stage				0.050*
1	8 (80%)	2 (20%)	10	
2	29 (42%)	40 (58%)	69	
3	22 (56.4%)	17 (43.6%)	39	
ER				0.038*
+	8 (40%)	12 (60%)	20	
−	36 (66.7%)	18 (33.3%)	54	
PR				0.089
+	10 (41.7%)	14 (58.3%)	24	
−	26 (63.4%)	15 (36.6%)	41	
Her2				0.863
+	8 (61.5%)	5 (38.5%)	13	
−	33 (58.9%)	23 (41.1%)	56	
Ki67				0.912
Low (≤50%)	34 (54%)	29 (46%)	63	
High (>50%)	1 (50%)	1 (50%)	2	

*ER* oestrogen receptors, *PR* progesterone receptors, *HER2* human epidermal growth factor receptor-2, *P < 0.05.

The Cancer Cell Line Encyclopedia (CCLE) database showed that the mRNA level of ZNF276 was higher in Luminal positive and HER2 positive breast cancer cells (Fig. 1G). Further validation of ZNF276 protein expression using six breast cancer cells with different molecular subtypes indicated that compared with MCF-10A, the protein levels of ZNF276 were upregulated in most breast cancer cells, but ZNF276 was undetectable in SK-BR-3 (Fig. 1H). Given that ZNF276 expression was not significantly correlated with estrogen receptor (ER) and progesterone receptor (PR) (Table 1), and ZNF276 did not show a significant expression characteristic in any specific subtype at the tissue and cellular levels (Fig. 1F–H), we speculated that ZNF276 expression was not significantly correlated with breast cancer subtypes. The Kaplan–Meier curve was drafted using data from TCGA public database and breast cancer tissue microarray with follow-up information. Both survival analysis showed that higher expression of ZNF276 indicated a lower overall survival of breast cancer patients (Fig. 1I, J). Altogether, these results indicate that ZNF276 is highly expressed in breast cancer tissues and cell lines.

### ZNF276 promotes the proliferation, invasion, and migration of breast cancer cells

**Fig. 2:**
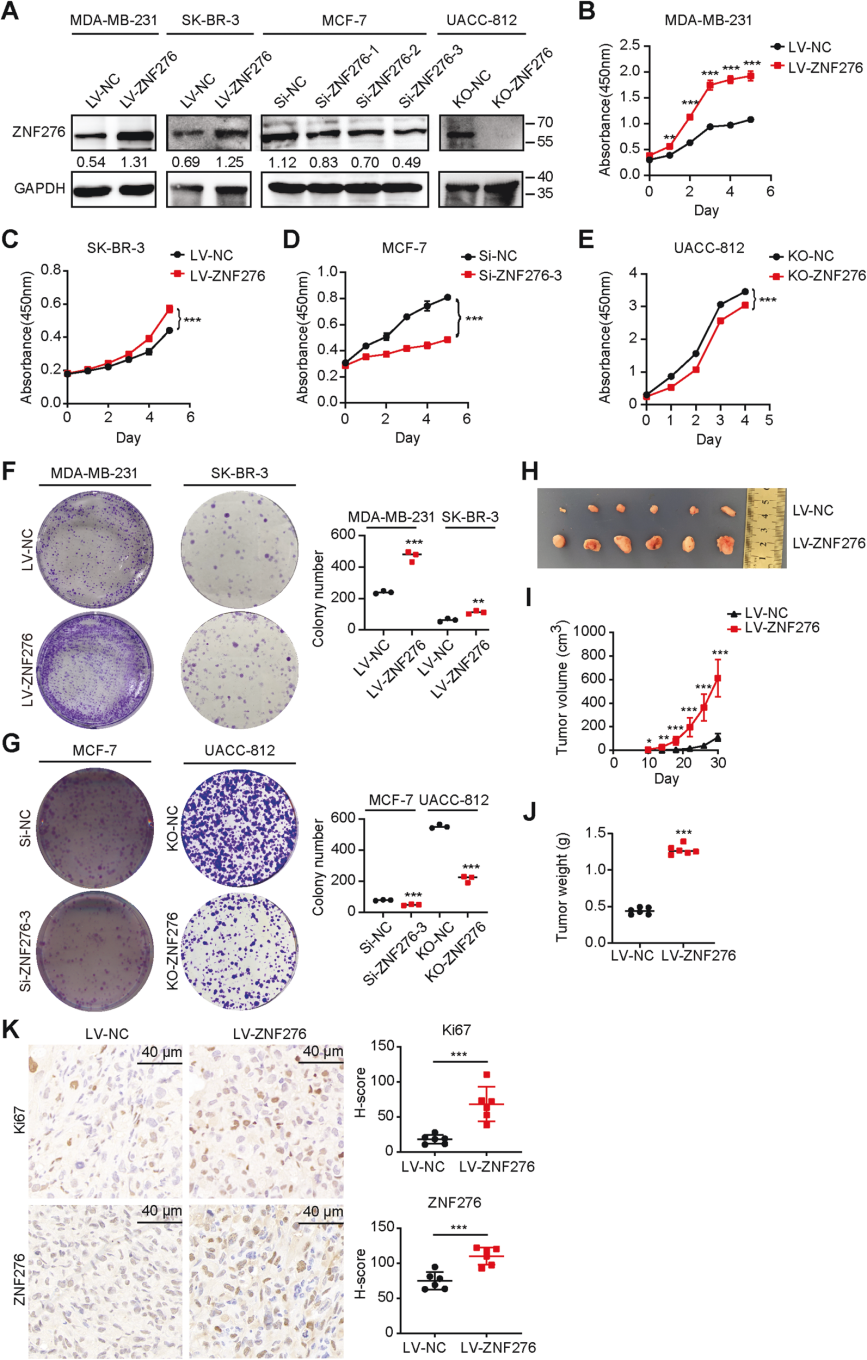
ZNF276 promotes the proliferation of breast cancer cells. **A** Western blot identifying the ZNF276 overexpression in MDA-MB-231 and SK-BR-3, ZNF276 knockdown in MCF-7 and ZNF276 knockout in UACC-812. B-C. CCK-8 assay measuring the effect of ZNF276 overexpression on cell proliferation in MDA-MB-231 (**B**) and SK-BR-3 (**C**) cells. **D**, **E** The effect of ZNF276 silencing (**D**) and knockout (**E**) on cell proliferation was evaluated by CCK-8 assay. **F** Cell proliferation ability of ZNF276 overexpressing cells was measured by clone formation assay. **G** Clone formation assay measuring the effect of ZNF276 silencing and knockout on cell proliferation. **H** ZNF276 overexpression affecting breast cancer tumor formation in the mammary fat pad of nude mice. **I** Measurement of tumor volume of the resected tumors from mice injected with ZNF276 overexpressing cells and control cells. **J** Statistical analysis of tumor weight of the resected tumors. **K** Immunohistochemical staining of Ki67 and ZNF276 in the resected tumors. Bar = 40 µm. *p < 0.05; **p < 0.01; ***p < 0.001.

As ZNF276 was overexpressed in breast cancer tissues and cells, we supposed that ZNF276 might play a role in breast cancer pathogenesis. Considering that the expression level of ZNF276 was highest in UACC-812 and MCF-7 cells, but lower in SK-BR-3 and MDA-MB-231 cells (Fig. 1H), we selected SK-BR-3 and MDA-MB-231 for ZNF276 overexpression, MCF-7 and MDA-MB-231 for ZNF276 knockdown and UACC-812 for ZNF276 knockout (Figs. 2A, S1A–F). A CCK-8 assay showed that overexpression of ZNF276 markedly enhanced the proliferation of MDA-MB-231 and SK-BR-3 cells (Fig. 2B, C), while a clone formation assay demonstrated that the colony-forming ability was notably increased after ZNF276 overexpression (Fig. 2F). Conversely, ZNF276 knockdown in MCF-7 and MDA-MB-231 cells and knockout in UACC-812 cells significantly weakened these abilities (Figs. 2D, E, G, S1G, H). To further investigate the pathological role of ZNF276 in tumorigenesis in vivo, we established xenograft models by injecting stable MDA-MB-231 cells or control cells into BALB/c nude mice. Thirty days after the tumor cell injection, the tumor weight and volume in nude mice with ZNF276-stably expressing cells were significantly larger and grew faster than those in the control group (Figs. 2H–J, S1I). There was a significantly increased level of tumor cell proliferation marker Ki67 in the ZNF276 overexpression group than in the control group (Fig. 2K).

**Fig. 3:**
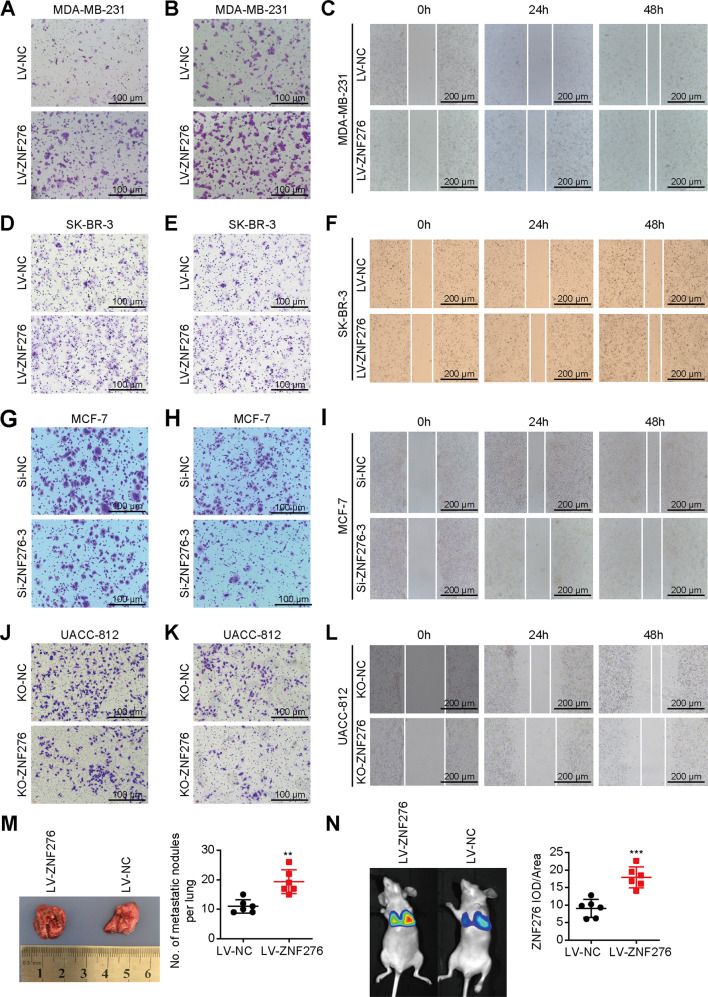
ZNF276 promoted cell migration and invasion. **A**, **B** The effect of ZNF276 overexpression on breast cancer cell migration (**A**) and invasion (**B**) detected by transwell assay in MDA-MB-231. Bar = 100 µm. **C** Migrated distance of MDA-MB-231 cells induced by ZNF276 transfection was measured from 0 h to 48 h. Bar = 200 µm. **D**–**F** The effect of ZNF276 overexpression on breast cancer cell migration and invasion were also measured in SK-BR-3 by transwell migration (**D**), transwell invasion (**E**) and wound healing assay (**F**). **G**, **H** Migration (**G**) and invasion (**H**) abilities of MCF-7 cells was reduced by ZNF276 knockdown using siRNA. Bar = 100 µm. **I** Migrated distance of MCF-7 cells induced by siZNF276 transfection was measured from 0 h to 48 h. Bar = 200 µm. **J**, **K** Migration (**J**) and invasion (**K**) abilities of UACC-812 cells was reduced by ZNF276 knockdown using sgRNA. Bar = 100 µm. **L** Cell migration of UACC-812 cells attenuated by sgZNF276 was determined by wound healing assay. Bar = 200 µm. **M** Comparison of the number of pulmonary metastatic nodules in mice injected with ZNF276 overexpressing cells and the control cells. **N** Bioluminescent Image analysis of mouse lungs dissected after necropsy. IOD, integral optical density. *p < 0.05; **p < 0.01; ***p < 0.001.

We then performed experiments associated with cell migration and invasion phenotypes in vitro. Transwell migration and matrigel invasion assays indicated that overexpression of ZNF276 increased the migrated and invasive abilities of MDA-MB-231 and SK-BR-3 cells (Figs. 3A, B, D, E, S2A, B, D, E). Besides, would healing assay demonstrated that overexpression of ZNF276 increased cell migration (Figs. 3C, F, S2C, F). In contrast, knockdown of ZNF276 by siRNA or sgRNA suppressed cell migration, invasion and wound closure speed in MCF-7, MDA-MB-231 and UACC-812 cells (Figs. 3G–L, SG–O). We also tested the pro-metastasis role of ZNF276 in a lung metastasis model. Mice bearing ZNF276-stably expressing cells formed more multiple metastasis nodules on the lung surface (Fig. 3M). Bioluminescent imaging showed that the average fluorescence intensity of overexpressed ZNF276 group was significantly higher than that of control group (Fig. 3N). In a word, ZNF276 could promote the proliferation, invasion and migration of breast cancer cells in vitro and in vivo.

### ZNF276 directly binds to the promoter of CYP1B1 and activates CYP1B1 expression

To explore the mRNA expression profile transcriptionally controlled by ZNF276, we performed RNA sequencing using MDA-MB-231 cells stably expressing ZNF276 and control cells. A total of 4 173 differentially expressed genes were screened according to the criteria of FC ≥ 2 and FDR < 0.05, including 2 194 upregulated genes and 1 979 downregulated genes (Fig. S3A, B). GO analysis revealed that the differentially expressed genes (DEG) were mainly associated with the G2/M transition of the mitotic cell cycle, perinuclear region of the cytoplasm and SH3 domain binding (Fig. S3C–E). KEGG analysis showed that DEG were mainly enriched in cancer-related pathways (Fig. S3F). We applied CUT&Tag for detection of ZNF276-DNA interaction status in breast cancer cells. A total of 4 432 genes with potential ZNF276 binding sites at promoter, 5’ UTR, 3’ UTR, exon, intron and distal intergenic region were screened (Fig. S4A). GO analysis showed that peak-associated genes were mainly enriched in the regulation of biological process, neuron part and phosphatidylinositol bisphosphate kinase activity, while KEGG analysis indicated that peak-associated genes were mainly associated with metabolic pathways (Fig. S4B, C). Motif analysis identified 25 expected ZNF276 binding sites motif present located from −250 to 250 bp upstream and downstream of the peak, with *GTGTGT* being the most significant motif (Fig. S4D).

**Fig. 4:**
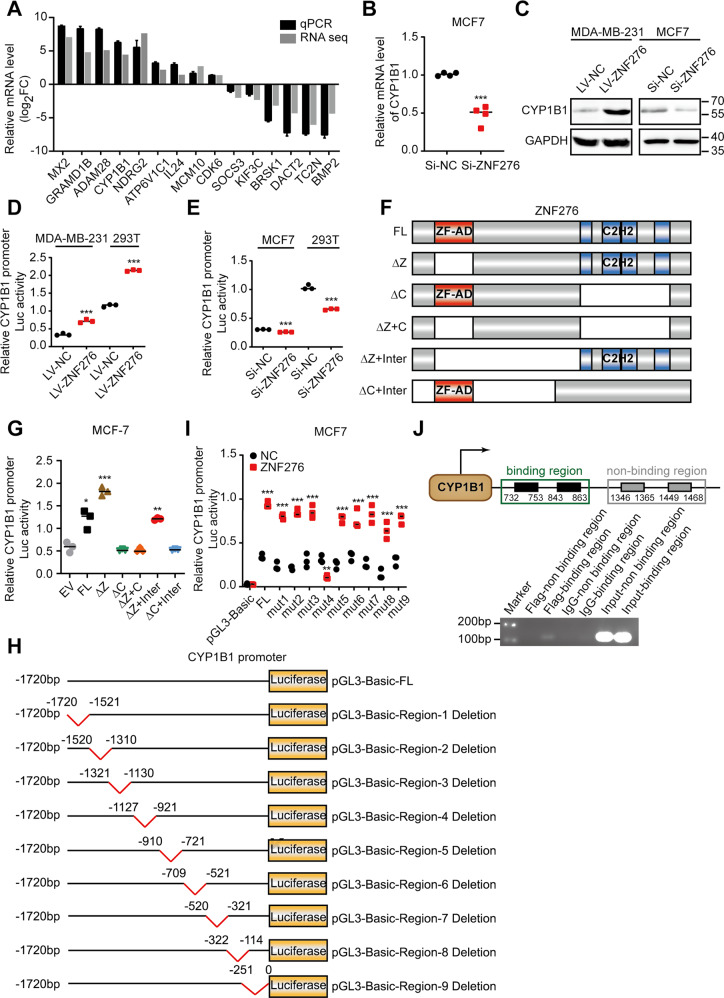
ZNF276 regulates CYP1B1 promoter transcriptional activity. **A** The mRNA levels of the 15 genes with relative high fold change were verified by RT-qPCR. **B** The effect of ZNF276 knockdown on the mRNA expression of CYP1B1. **C** Western blot analysis of the effect of ZNF276 overexpression and knockdown on CYP1B1 expression. **D** Dual-luciferase reporter gene assay showing ZNF276 overexpression promoted luciferase activity of CYP1B1 promoter. **E** Dual-luciferase reporter gene assay showing inhibition of ZNF276 decreased luciferase activity of CYP1B1 promoter. **F** Construction of truncated fragments of ZNF276 for investigating the binding site of ZNF276 on CYP1B1 promoter. **G** Dual-luciferase reporter gene assay measuring the CYP1B1 promoter luciferase activity driven by different truncated fragment of ZNF276. **H** Construction of truncated fragments of CYP1B1 promoter for investigating the ZNF276 binding site. **I** Dual-luciferase reporter gene assay measuring the truncated CYP1B1 promoter luciferase activity in ZNF276 overexpressing and control cells. **J** ChIP-PCR assay demonstrated a direct binding of ZNF276 at the CYP1B1 promoter. *p < 0.05; **p < 0.01; ***p < 0.001.

Genes that might be transcriptionally controlled by ZNF276 and important for ZNF276-mediated cell proliferation and metastasis were selected for study in the flowchart in Figure S4E. We chose 15 genes of interest from the overlapped genes between RNA-seq and CUT&Tag (Table S3), and validated their expressions by RT-qPCR in independent samples. The expression trend was consistent with the results from RNA-sequencing data (Fig. 4B). We were particularly interested in CYP1B1 due to the high fold change (log_2_FC = 4.45) and peak enrichment (fold enrichment=5.08). The expression of CYP1B1 was significantly higher in tumor tissues than in normal tissues, such as breast, ovarian and prostate tumors [15–17], and CYP1B1 functions as an oncogene that promotes tumor progression in multiple tumors [18, 19]. Moreover, there were reports indicating that CYP1B1 could promote cell proliferation and metastasis by activating Wnt/β-catenin pathway [9, 10]. Therefore, we chose CYP1B1 as the downstream target of ZNF276 for the following study.

Overexpression of ZNF276 elevated CYP1B1 expression at mRNA and protein levels, while knockdown of ZNF276 inhibited CYP1B1 expression (Fig. 4C, D). Dual-luciferase assay demonstrated that overexpression of ZNF276 significantly enhanced the transcriptional activity of the CYP1B1 promoter, while knockdown of ZNF276 reduced it (Fig. 4E, F). ChIP-PCR analysis in MDA-MB-231 cells provided more definitive evidence that ZNF276 directly bond to the promoter of CYP1B1 (Fig. 4G). We further explored the functional CYP1B1 promoter binding domain within ZNF276. As shown in the InterPro website (https://www.ebi.ac.uk/interpro/protein/UniProt/Q8N554/), ZNF276 contains the ZF-AD (zinc finger associated domain) and C_2_H_2_ domains. We constructed five plasmids with mutations in the ZF-AD and C_2_H_2_ domains of ZNF276 (Fig. 4H). No significant differences were observed in CYP1B1 promoter activity between the ZNF276∆C, ZNF276∆Z + C and ZNF276∆C + Inter groups and the control group, except for all three groups with mutated C_2_H_2_ domains, indicating that ZNF276 binds to the CYP1B1 promoter via the C_2_H_2_ domain (Fig. 4I). We also designed and constructed nine truncated fragments within the promoter of CYP1B1, each of which had a length of approximately 200 bp (Fig. 4J). We found that ZNF276 failed to activate CYP1B1 promoter activity when the −1127 to −921 bp region of the CYP1B1 promoter was deleted (Fig. 4K). Consequently, ZNF276 binds to the −1127 to −921 bp region of the CYP1B1 promoter through its C_2_H_2_ domain, thereby transcriptionally regulating CYP1B1 expression.

### ZNF276 activates the Wnt/β-catenin signaling pathway by regulating CYP1B1 expression

**Fig. 5:**
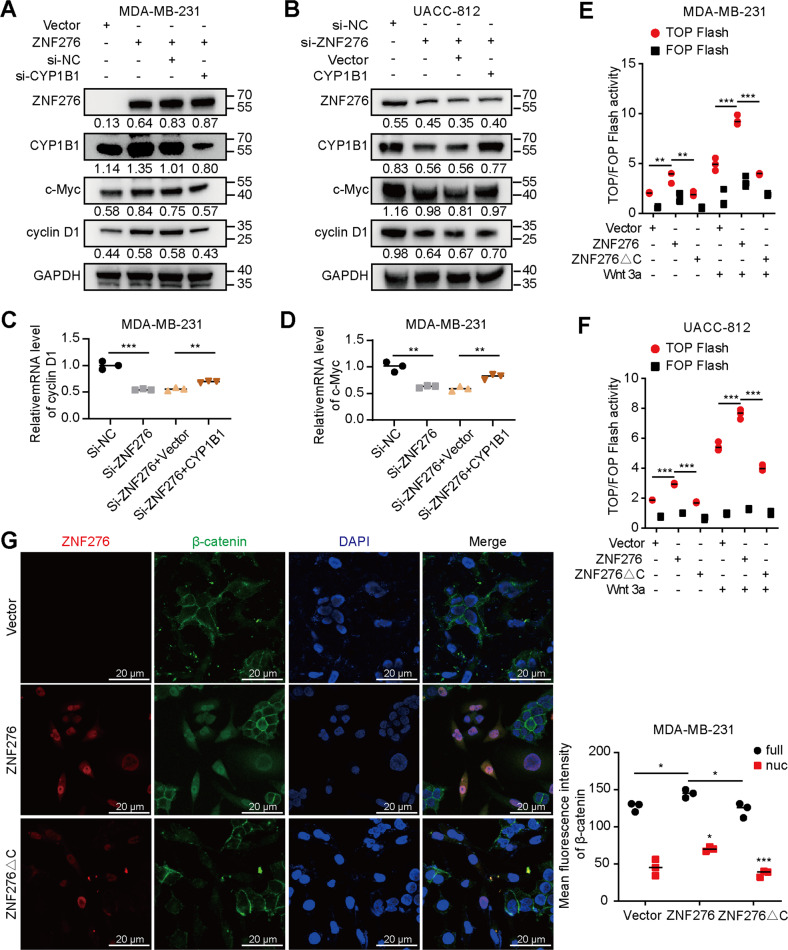
ZNF276 activates the Wnt/β-catenin signaling by regulation of CYP1B1. **A**, **C** Western blotting (**A**) and RT-qPCR (**C**) analysis revealed that silencing of CYP1B1 inhibited the expression of c-Myc and Cyclin D1 enhanced by ZNF276 overexpression in MDA-MB-231 cells. **B**, **D** Effects of ZNF276 knockdown and CYP1B1 overexpression on c-Myc and Cyclin D1 expression were analyzed in UACC-812 cells by western blotting (**B**) and RT-qPCR (**D**). **E**, **F** ZNF276 lacking C_2_H_2_ domain inhibited the TOP Flash activity enhanced by the intact ZNF276 with wnt3a activation in MDA-MB-231 (**E**) and UACC-812 (**F**) cells. **G** Endogenous β-catenin expression and distribution was observed by immunofluorescence in MDA-MB-231 cells transfected with intact ZNF276 or ZNF276 lacking C_2_H_2_ domain plasmid. Nuc nuclear. Bar = 20 µm. *p < 0.05; **p < 0.01; ***p < 0.001.

Considering the correlation between CYP1B1 and Wnt/β-catenin pathway, we speculated that ZNF276 may affect the activation of Wnt/β-catenin pathway by regulating CYP1B1 expression. Firstly, CYP1B1 expression regulated by transient overexpression or specific siRNAs was verified using western blotting (Fig. S5A). Overexpression of ZNF276 promoted the protein expressions of downstream targets, c-Myc and cyclin D1, which were partially attenuated by knockdown of CYP1B1 (Fig. 5A). In contrast, knockdown of ZNF276 inhibited the protein levels of c-Myc and cyclin D1, which were reversed by CYP1B1 overexpression (Fig. 5B). A similar expression trend of c-Myc and Cyclin D1 was also observed at mRNA levels by RT-qPCR analysis (Fig. 5C, D). The TOP/FOP Flash assay could reflect Wnt/β-catenin transcriptional activity. Overexpression of ZNF276 in MDA-MB-231, MCF-7, UACC-812 and 293T cells increased the TOP Flash activity, with Wnt3a stimulation further increasing the signal, but ZNF276 expression had no effect on FOP Flash activity. ZNF276 lacking C_2_H_2_ domain was unable to increase TOP Flash activity in 293T and MCF-7 cells, indicating that ZNF276 activates the Wnt/β-catenin pathway by binding to the CYP1B1 promoter and increasing its expression (Figs. 5E, F, S5A, B). The subcellular localization of β-catenin affected by ZNF276 overexpression was detected in breast cancer cells using immunofluorescence. Surprisingly, overexpression of ZNF276 not only promoted β-catenin expression, but also its translocation from cytoplasm to nucleus, whereas ZNF276 lacking C_2_H_2_ domain failed to affect the expression and cytoplasm/nucleus distribution of β-catenin (Fig. 5I).

Collectively, these data suggested that ZNF276 activates the Wnt/β-catenin pathway by promoting CYP1B1-mediated β-catenin nuclear translocation and downstream target genes expression.

### CYP1B1 is the functional target of ZNF276

**Fig. 6:**
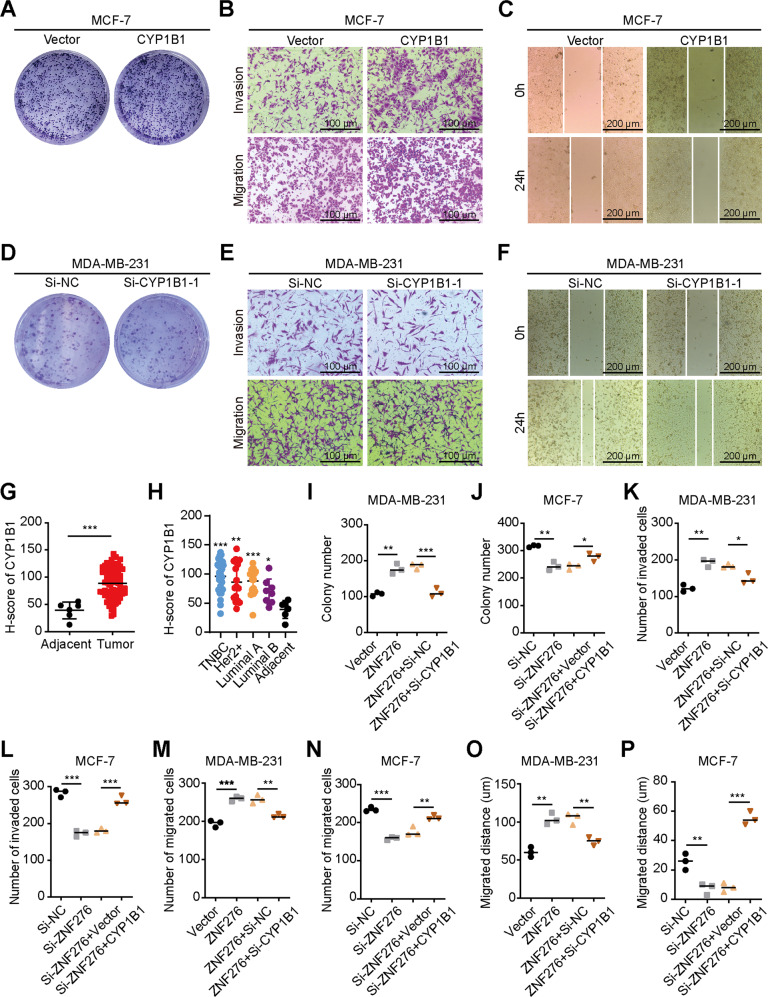
CYP1B1 is the functional target of ZNF276. **A** The clone formation assay was used to detect the effect of CYP1B1 on cell proliferation. **B** Migration and invasion abilities affected by CYP1B1 overexpression were measured by transwell migration and invasion assay. Bar = 100 µm. **C** Migrated distance was calculated in CYP1B1 overexpressing and control cells in wound healing assay. Bar = 200 µm. **D** The colony formation assay was performed with MDA-MB-231 cells transfected with si-CYP1B1 or si-NC. **E**, **F** The effect of CYP1B1 knockdown on migration and invasive abilities of MDA-MB-231 cells were measured by transwell assay (**E**) and wound healing assay (**F**). **G** Analysis of CYP1B1 protein expression in breast cancer tissues and adjacent tissues by immunohistochemistry. **H** The protein expression of CYP1B1 in different molecular subtypes of breast cancer samples was detected by immunohistochemistry. **I** Silencing of CYP1B1 partly reversed the effect of ZNF276 on cell clone formation. **J** Overexpression of CYP1B1 promoted cell clone formation ability that was inhibited by ZNF276 knockdown. **K** Silencing of CYP1B1 neutralized the effect of ZNF276 on cell invasion. **L** Overexpression of CYP1B1 reversed cell invasion ability downregulated by ZNF276 inhibition. **M** Silencing of CYP1B1 neutralized the effect of ZNF276 on cell migration. **N** Overexpression of CYP1B1 reversed cell migration ability downregulated by ZNF276 inhibition. **O** Wound healing assay indicating that the effect of ZNF276 on cell migration was partly reversed by CYP1B1 knockdown. **P** The expression of CYP1B1 enhanced cell migration ability attenuated by ZNF276. *p < 0.05; **p < 0.01; ***p < 0.001.

As the role of CYP1B1 in breast cancer was still unknown, we first explored the effect of CYP1B1 on breast cancer cells proliferation, migration and invasion. Overexpression of CYP1B1 promoted MCF-7 cell proliferation, invasion and migration, which was similar to the effects of ZNF276 upregulation (Figs. 6A–C, S5A–C). CYP1B1 knockdown inhibited MDA-MB-231 cell proliferation, invasion and migration, which was consistent with the effects of ZNF276 downregulation (Figs. 6D–F, S5D–F). In addition, immunohistochemistry confirmed that CYP1B1 was highly expressed in breast cancer tissues (Figs. 6G, H, S5G), indicating that CYP1B1 plays an oncogenic role on breast cancer progression.

We then hypothesized that CYP1B1 could be responsive for the effects of ZNF276 on the malignant phenotypes of breast cancer cells. CYP1B1 was downregulated in MDA-MB-231 cells with ZNF276 overexpression and upregulated in MCF-7 cells with ZNF276 silencing (Fig. S5H, I). Knockdown of CYP1B1 partially attenuated the ZNF276 overexpression-enhanced proliferation, migration and invasion of MDA-MB-231 cells, while CYP1B1 overexpression reversed the ZNF276 knockdown-mediated inhibitory effect on the proliferation, migration and invasion of MCF-7 cells (Figs. 6I–P, S6). Taken together, these results demonstrated that CYP1B1 is the functional target of ZNF276 in breast cancer.

### MAGEB2 interacted with ZNF276 to facilitate the expression of CYP1B1 and Wnt/β-catenin signaling activation

**Fig. 7:**
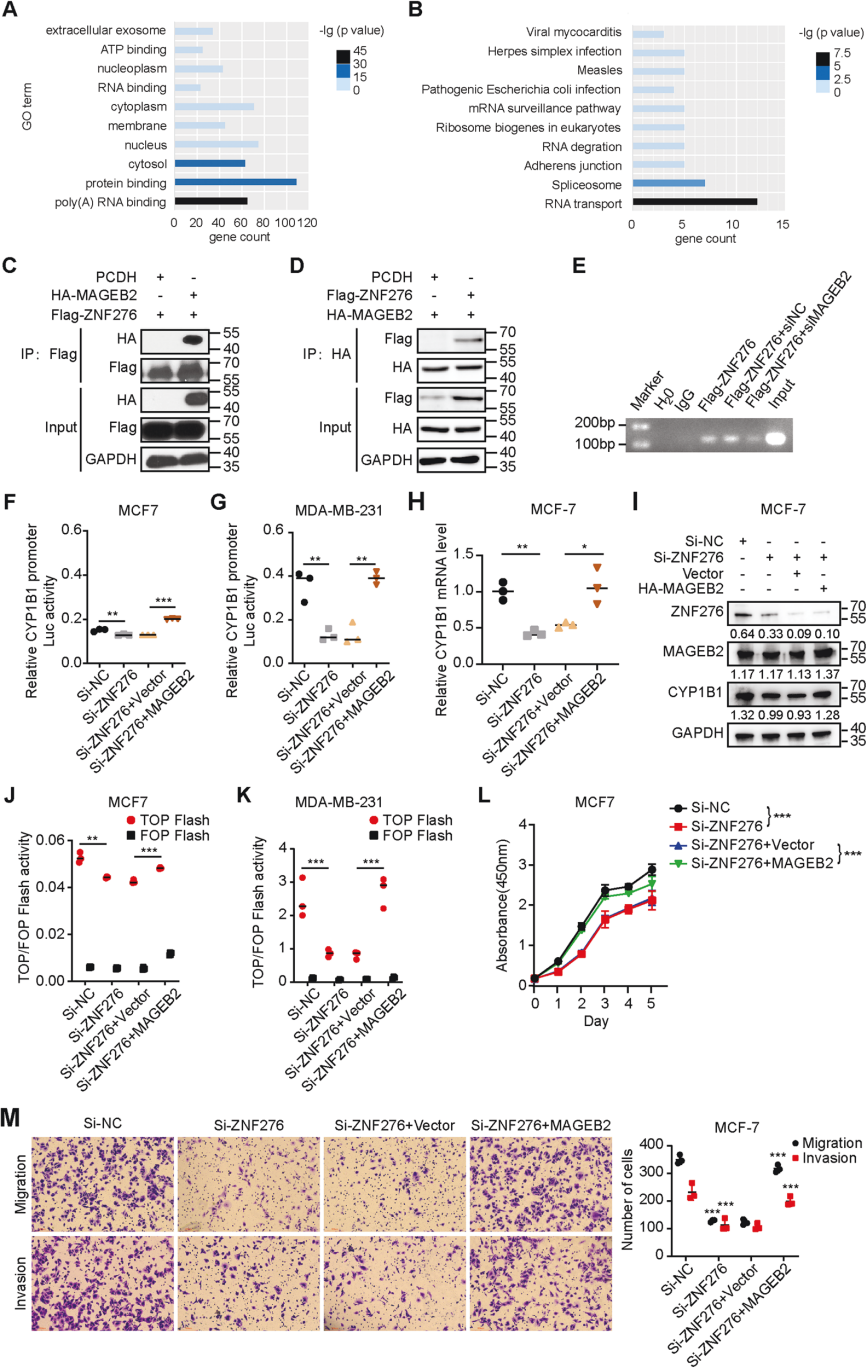
ZNF276 activates Wnt/β-catenin signaling through recruitment of MAGEB2 to regulate CYP1B1 transcription. **A** GO analysis of the potential ZNF276-interacted proteins. **B** KEGG analysis of the potential ZNF276-interacted proteins. **C**, **D** Both forward (**C**) and reverse (**D**) co-IP analysis affirmed the interaction between ZNF276 and MAGEB2. **E** ChIP-PCR analysis of ZNF276 binding ability at the CYP1B1 promoter affected by MAGEB2 overexpression. **F**, **G** The luciferase activity of CYP1B1 promoter decreased by ZNF276 silencing was further enhanced by the expression of MAGEB2 in 293T and MCF-7. **H**, **I** RT-qPCR (**H**) and western blot (**I**) analysis confirmed the effect of MAGEB2 overexpression in CYP1B1 mRNA and protein expression in MCF-7 cells. **J**, **K** The reduced transcriptional activity of CYP1B1 promoter by ZNF276 was partially reversed by MAGEB2. **L**, **M** Overexpression of MAGEB2 reversed the decreased cell proliferation, migration and invasion abilities affected by ZNF276 silencing in CCK-8 assay (**L**), transwell migration (**M**, up) and transwell invasion (**M**, down).

To further explore the molecular mechanism underlying how ZNF276 regulated the expression of CYP1B1 transcription, we performed immunoprecipitation (IP) plus mass spectrometry to profile the ZNF276 interactome. After removing the non-specific binding protein from the control group, a total of 1 001 proteins were considered as the potential ZNF276 binding proteins. Functional annotation and enrichment analysis for proteins with unique peptides≥3 was performed using DAVID. GO analysis showed that these proteins were mainly associated with the cytosol, protein binding and poly(A) RNA binding (Fig. 7A). KEGG analysis revealed that these proteins were mainly enriched in RNA transport, the spliceosome and adherens junction pathways (Fig. 7B).

Among all ZNF276 interacting proteins, MAGEB2 (unique peptide = 10) attracted our most attention. MAGEB2 has been reported to be overexpression in several types of carcinomas [11, 12], which has been implicated in carcinogenesis and identified as a potential cancer biomarker [12]. MAGEB2 also played an important role in the proliferation and metastasis of tumors [20]. Moreover, given that the tumor-associated protein MAGEB2 with the ten unique peptides could enhance E2F transcription factor activity by interacting with HDAC [20], we selected MAGEB2 as the candidate interacting protein for ZNF276. A bidirectional co-IP assay confirmed the interaction between ZNF276 and MAGEB2 (Fig. 7C, D). Knockdown of MAGEB2 weakened the binding activity of ZNF276 at the CYP1B1 promoter (Fig. 7E). MAGEB2 partially restored the inhibitory effect on CYP1B1 promoter transcription mediated by ZNF276 knockdown (Fig. 7F, G). RT-qPCR and western blot further confirmed that expression of MAGEB2 could reverse the mRNA and protein expression of CYP1B1 reduced by ZNF276 knockdown (Fig. 7H, I). The decreased TOP Flash activity in 293T and MCF-7 cells mediated by ZNF276 knockdown could be partially reversed by MAGEB2 overexpression (Fig. 7J, K). We investigated the role of MAGEB2 in ZNF276-mediated cell proliferation, migration and invasion. Overexpression of MAGEB2 significantly recovered the proliferation and migration abilities of breast cancer cell with ZNF276 silencing (Fig. 7L, M). Therefore, ZNF276 could activate the Wnt/β-catenin pathway by recruiting MAGEB2 to regulate CYP1B1 promoter transcription.

## Discussion

ZNF276, also called ZNF477 or CENP-Z, was originally discovered in a mouse model of Fanconi anemia by Wong et al. [21]. ZNF276 is located on chromosome 16q24.3, where loss of heterozygosity (LOH) frequently occurs and its encoding protein contains five C_2_H_2_ domains [5]. In this study, we found that ZNF276 was upregulated in both breast cancer tissues and cell lines. However, there was no significant correlation between ZNF276 expression and breast cancer subtypes. Moreover, overexpression of ZNF276 accelerated cell proliferation, migration and invasion, indicating that ZNF276 functions as an oncogene in breast cancer. Generally, higher ZNF protein expression correlated with the tumor-promoting effect. ZEB1 is overexpressed in breast cancer, glioma and pancreatic cancer, induces epithelial-mesenchymal transition (EMT) and promotes tumor invasion [22–24]. ZNF224 showed higher expression in bladder cancer, liver cancer and breast cancer and acted as an oncogene by recruiting DEPDC1 to form a transcription complex. Interestingly, ZNF224 expression was lower in chronic granulocytic leukemia and exhibited proapoptotic and antiproliferative effects with the ancillary transcription factor WT1 [25]. Due to the diversity of transcription regulation patterns, it is possible for the same ZNF proteins to play different roles in different cancers. Therefore, how ZNF276 functions in other cancers still requires exploration.

ZNF proteins are required to recruit several coactivators to control downstream gene expression. In this study, we demonstrated that ZNF276 could bind to and recruit MAGEB2 to facilitate CYP1B1 transcription and Wnt/β-catenin pathway activation. MAGEB2 is a proliferation-associated oncogene. MAGEB2 could interact with HDAC to promote the transcriptional activation of E2F, the effect of which was p53-independent [20]. Nucleolar MAGEB2 could also recruit phospho-upstream binding factor (pUBF) to the promoter of rDNA to activate rDNA transcription, accelerate ribosome biosynthesis and promote cancer cell proliferation [26]. Genome-wide analysis in head and neck squamous cell carcinoma revealed that MAGEB2 is activated by promoter demethylation and demonstrates a growth-promoting effect in a minimally transformed oral keratin-forming cell line [13]. Given that MAGEB2 is associated with promoter demethylation, we speculate that ZNF276 may activate transcription by recruiting MAGEB2 to demethylate the CYP1B1 promoter, which may provide ideas for subsequent studies.

CYP1B1 is an E2 oestrogen hydroxylase that belongs to the cytochrome P450 1 superfamily [27]. The expression of CYP1B1 in tumor tissues was considerably higher than that in normal tissues, especially in hormone-related cancers, such as breast cancer, ovarian cancer, glioma and prostatic cancer [28]. CYP1B1 could also enhance the expression of Sp1, thereby activating the Wnt/β-catenin pathway and EMT and promoting cell proliferation and metastasis [10]. Although CYP1B1 could promote the translocation of β-catenin from the cytoplasm to the nucleus, how CYP1B1 drives this process is still unknown. A recent study showed that overexpression of CYP1B1 could inhibit the expression of HERC5 and ISG15, two key factors in ISGylation [29]. CYP1B1 also interacted with HERC5. ISGylation, similar to SUMOylation, works by initiating the signaling cascade of E1 (Ube1L), E2 (UbcH8) and E3 (HERC5) ligases. Interferon-stimulated gene 15 (ISG15) is an IFN α/β-induced ubiquitin-like protein that is necessary for HERC5 conjunction [30]. The conserved C-terminus of ISG15 could recognize and bind to the target protein. Based on the above evidence, we hypothesized that CYP1B1 could bind to HERC5 and possibly inhibit HERC5-induced ISGylation of β-catenin, leading to the accumulation of β-catenin in the cytoplasm and translocation into the nucleus.

β-Catenin can be phosphorylated by GSK3β [31]. Phosphorylated β-catenin is degraded by the β-TrCP-mediated ubiquitin-proteasome pathway. Unphosphorylated β-catenin can translocate into the nucleus, bind to TCF/LEF [32, 33], recruit coactivators, such as CBP/p300 [34], pygopus [35] and BCL9 [36], and promote the transcription of Wnt-target genes [17]. In addition to GSK3β, the phosphorylation of β-catenin can also be affected by EGFR and FGFR [37]. We hypothesized that ZNF276-mediated CYP1B1 activation and β-catenin translocation may represent a novel mechanism for the Wnt/β-catenin pathway. However, this pathway was not the only pathway that could be significantly regulated by ZNF276 and lead to ZNF276-mediated cell proliferation, migration and invasion. ZNF276 levels were also significantly correlated with the cell cycle and metabolic pathways based on the RNA sequencing and CUT&Tag results in our study. Therefore, ZNF276-related mechanisms and downstream regulatory networks are worthy of further attention and exploration.

In summary, this is the first study describing the expression of ZNF276 in breast cancer tissues and cells and elucidating a novel mechanism by which ZNF276 promotes cell proliferation, migration and invasion through activation of the Wnt/β-catenin pathway. This study broadened our understanding of ZNF family proteins in malignancies and might provide novel strategies for Wnt/β-catenin pathway-related therapy.

## Supplementary information

reproducibility checklist

Figure S1

Figure S2

Figure S3

Figure S4

Figure S5

Figure S6

Figure S7

Supplementary Figure Legends

Original Data File 1

Original Data File 2

Table S1

Table S2

Table S3

## Data Availability

The datasets and materials used and/or analyzed during the current study are available from the corresponding author on reasonable request.
