# Supplementary figures and images for: ZNF276 promotes the malignant phenotype of breast carcinoma by activating the CYP1B1-mediated Wnt/β-catenin pathway
Source: Cell Death Dis. 2022 Sep 10;13(9):781. doi: 10.1038/s41419-022-05223-8 (PMC9463175; doi:10.1038/s41419-022-05223-8)

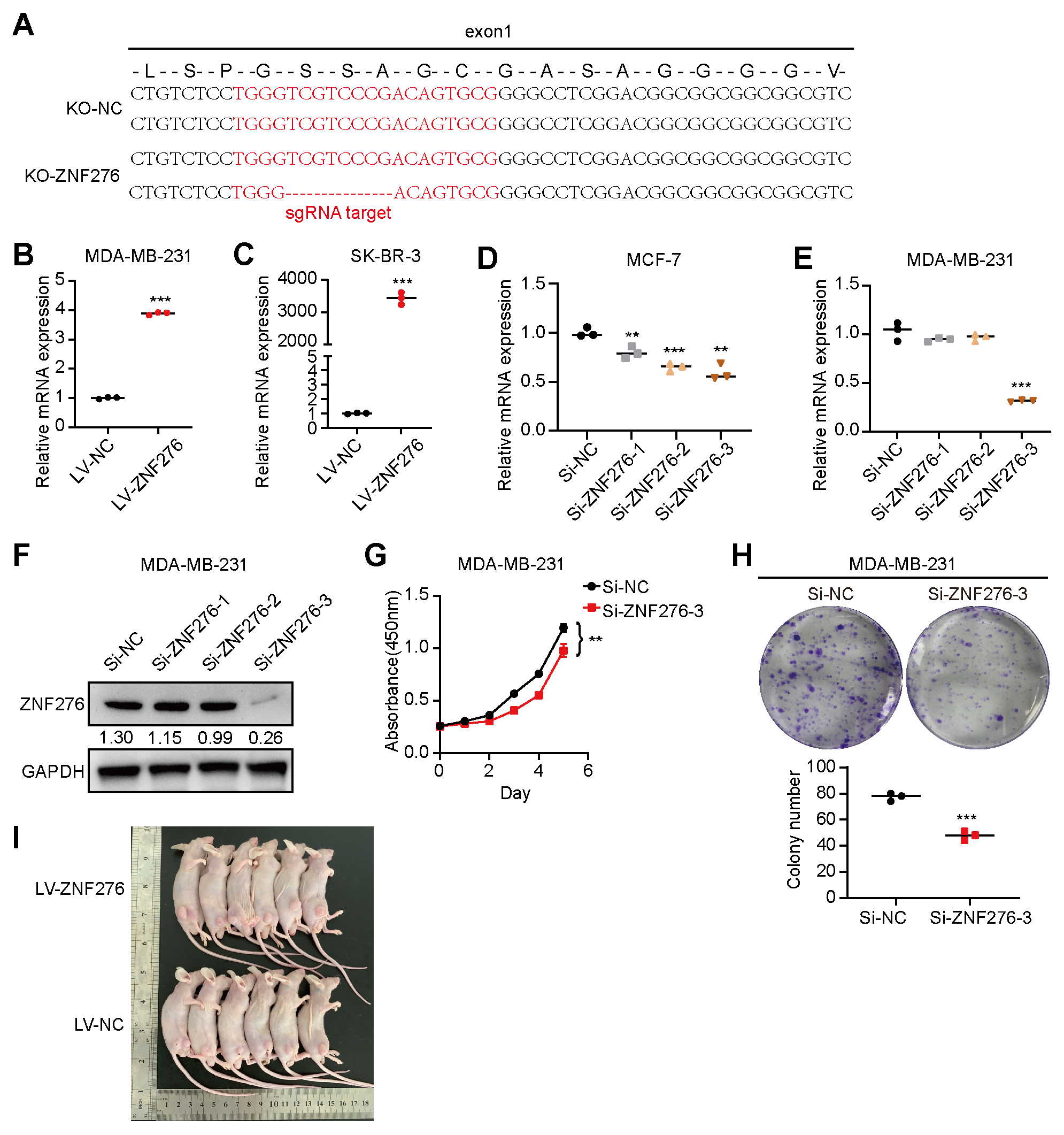

Supplement: Supplementary file 2 — Figure S1 [file 41419_2022_5223_MOESM2_ESM.tif]

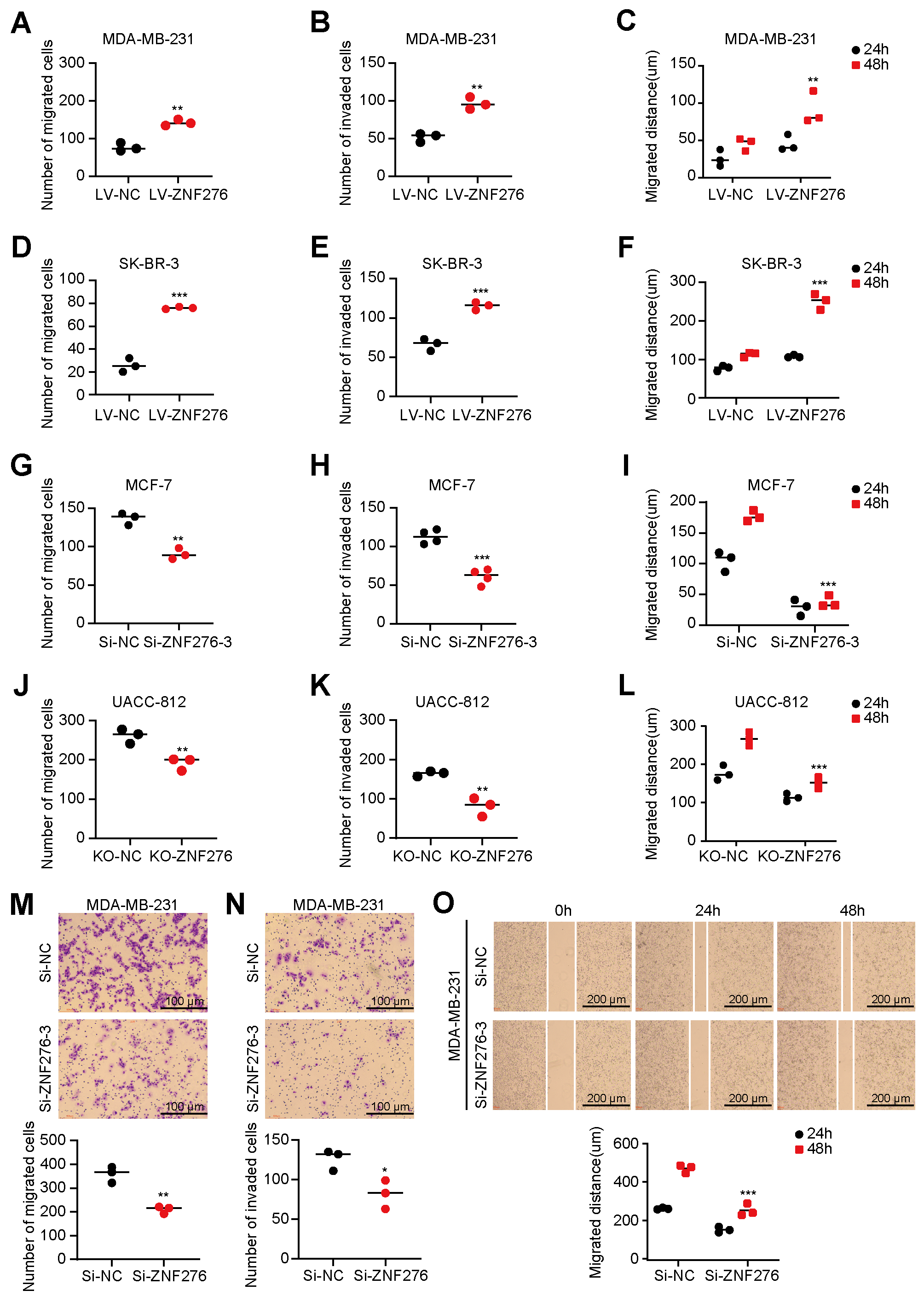

Supplement: Supplementary file 3 — Figure S2 [file 41419_2022_5223_MOESM3_ESM.tif]

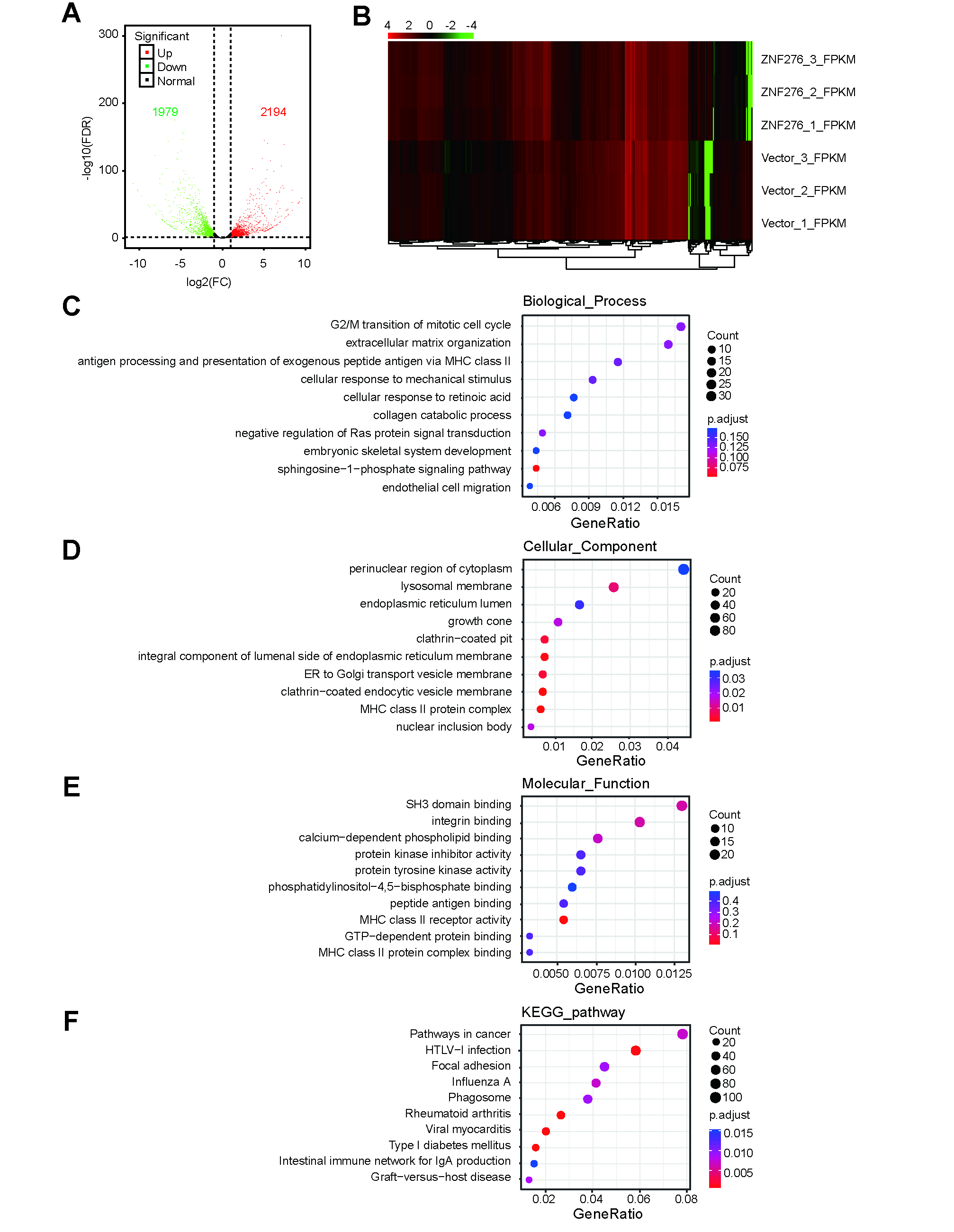

Supplement: Supplementary file 4 — Figure S3 [file 41419_2022_5223_MOESM4_ESM.tif]

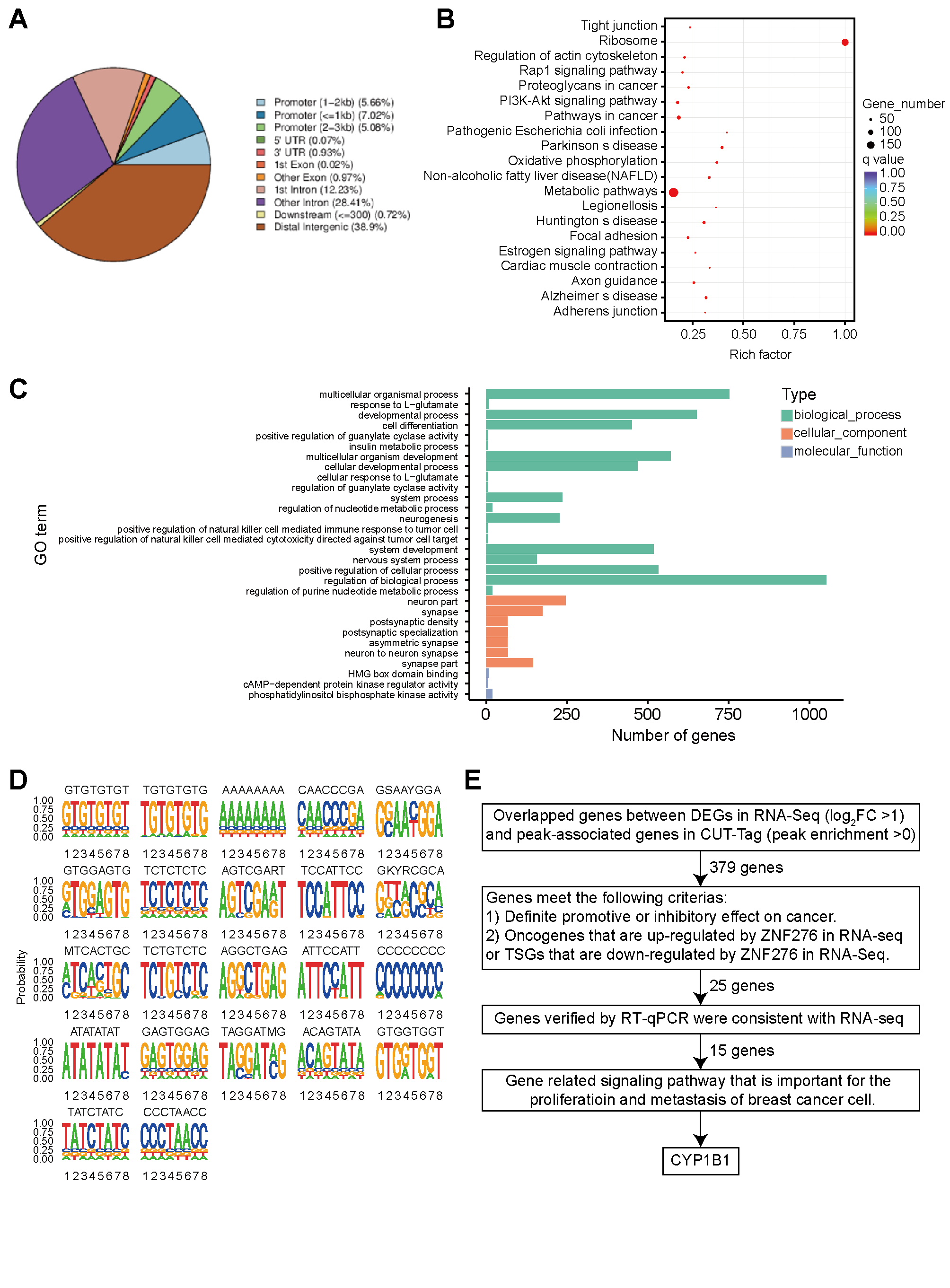

Supplement: Supplementary file 5 — Figure S4 [file 41419_2022_5223_MOESM5_ESM.tif]

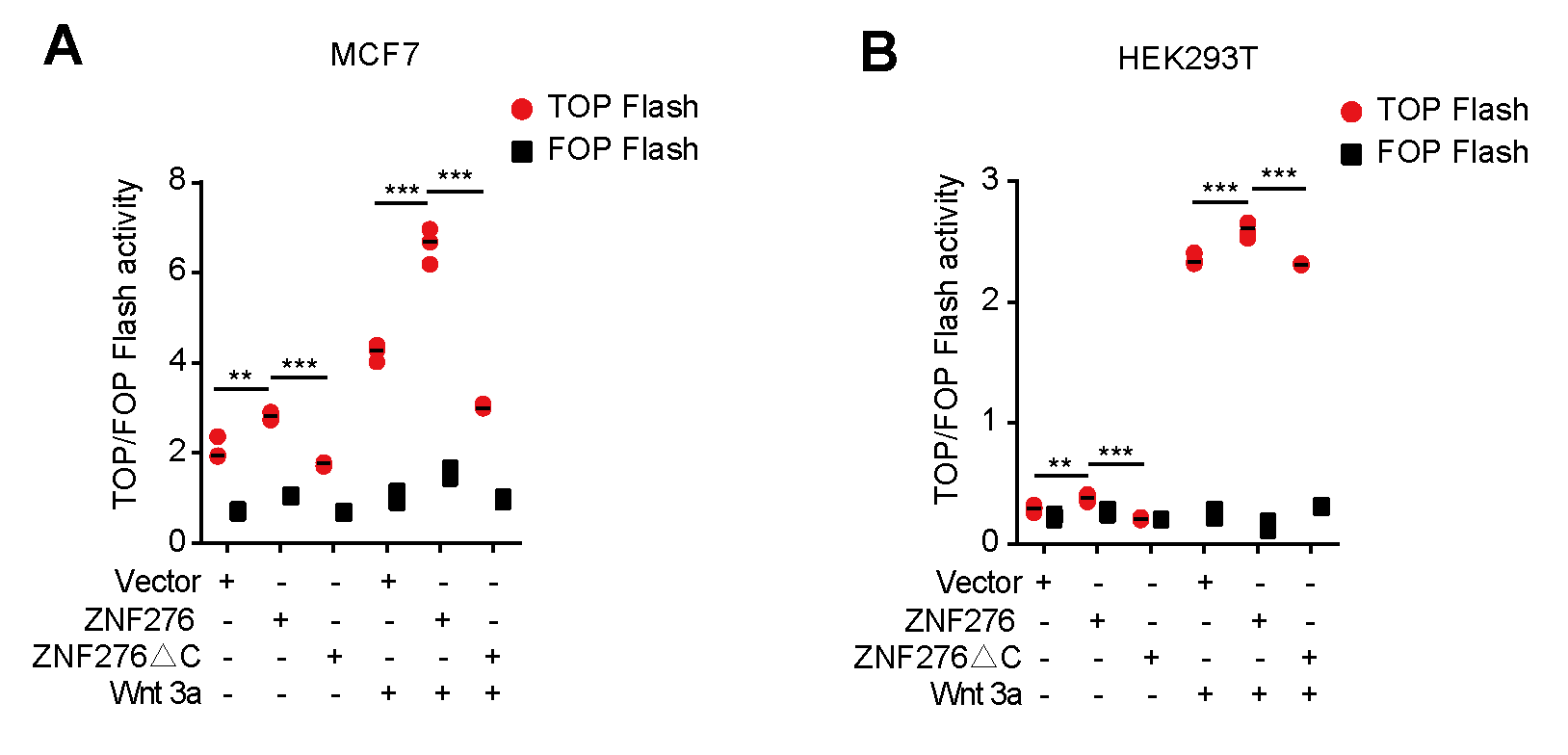

Supplement: Supplementary file 6 — Figure S5 [file 41419_2022_5223_MOESM6_ESM.tif]

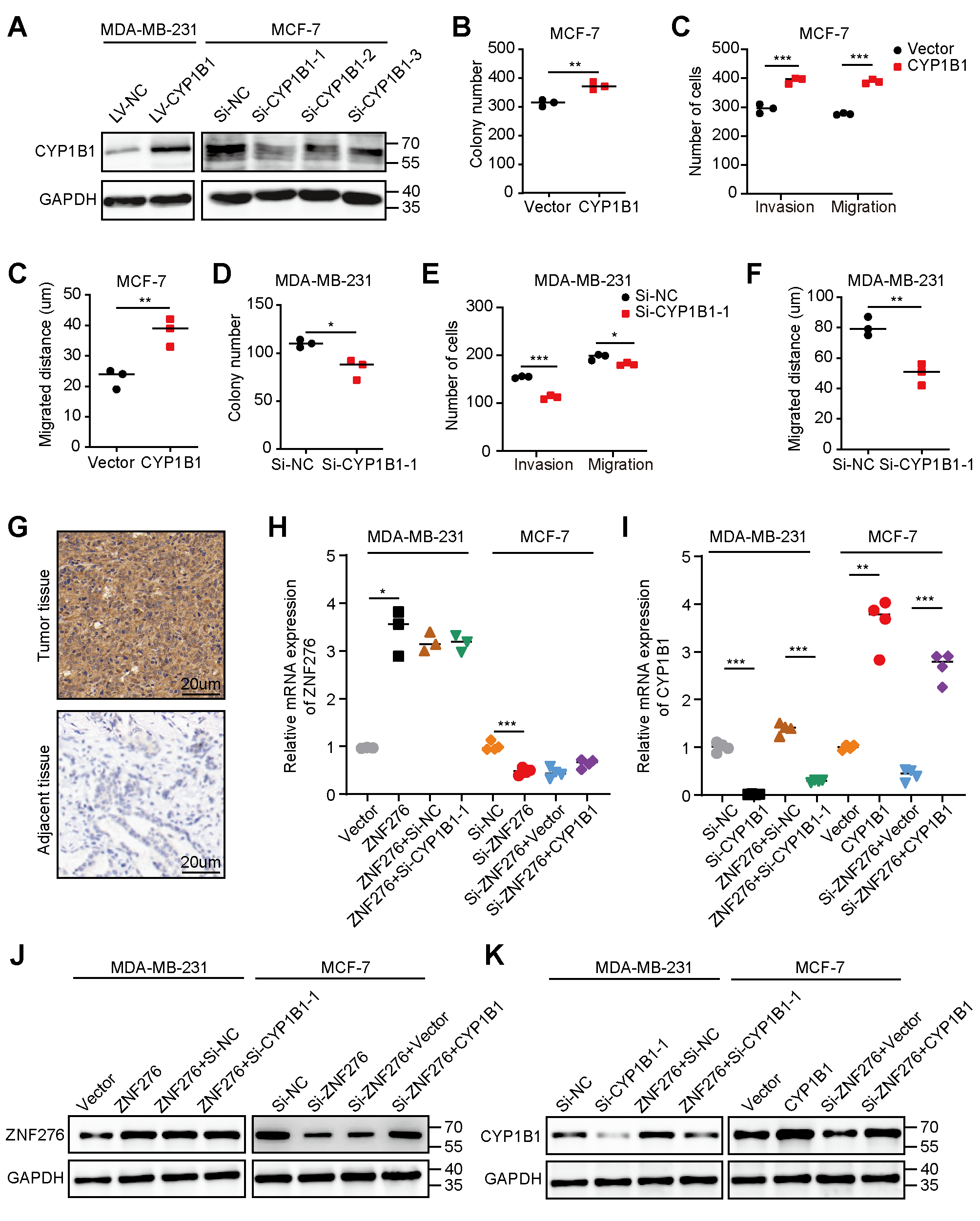

Supplement: Supplementary file 7 — Figure S6 [file 41419_2022_5223_MOESM7_ESM.tif]

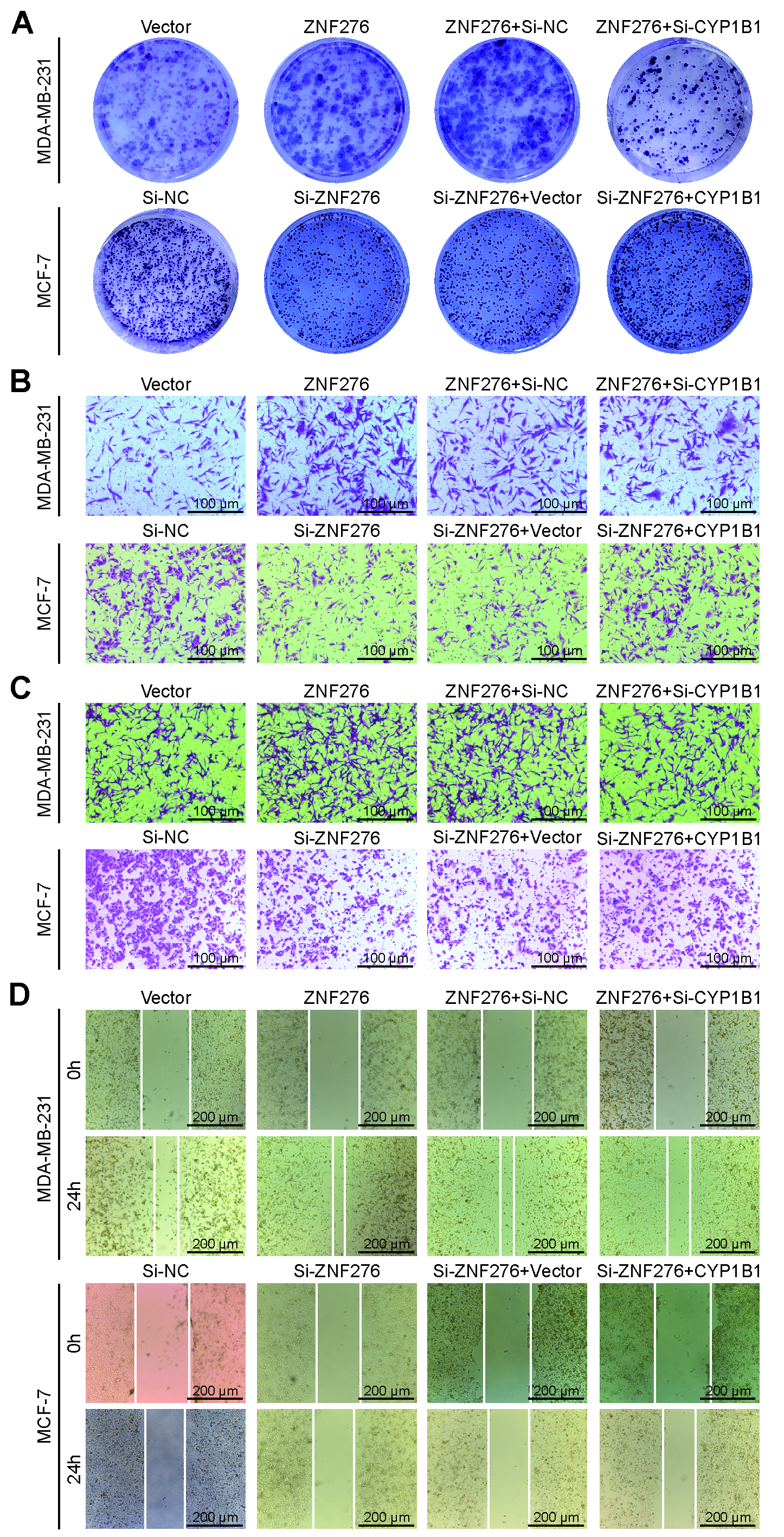

Supplement: Supplementary file 8 — Figure S7 [file 41419_2022_5223_MOESM8_ESM.tif]

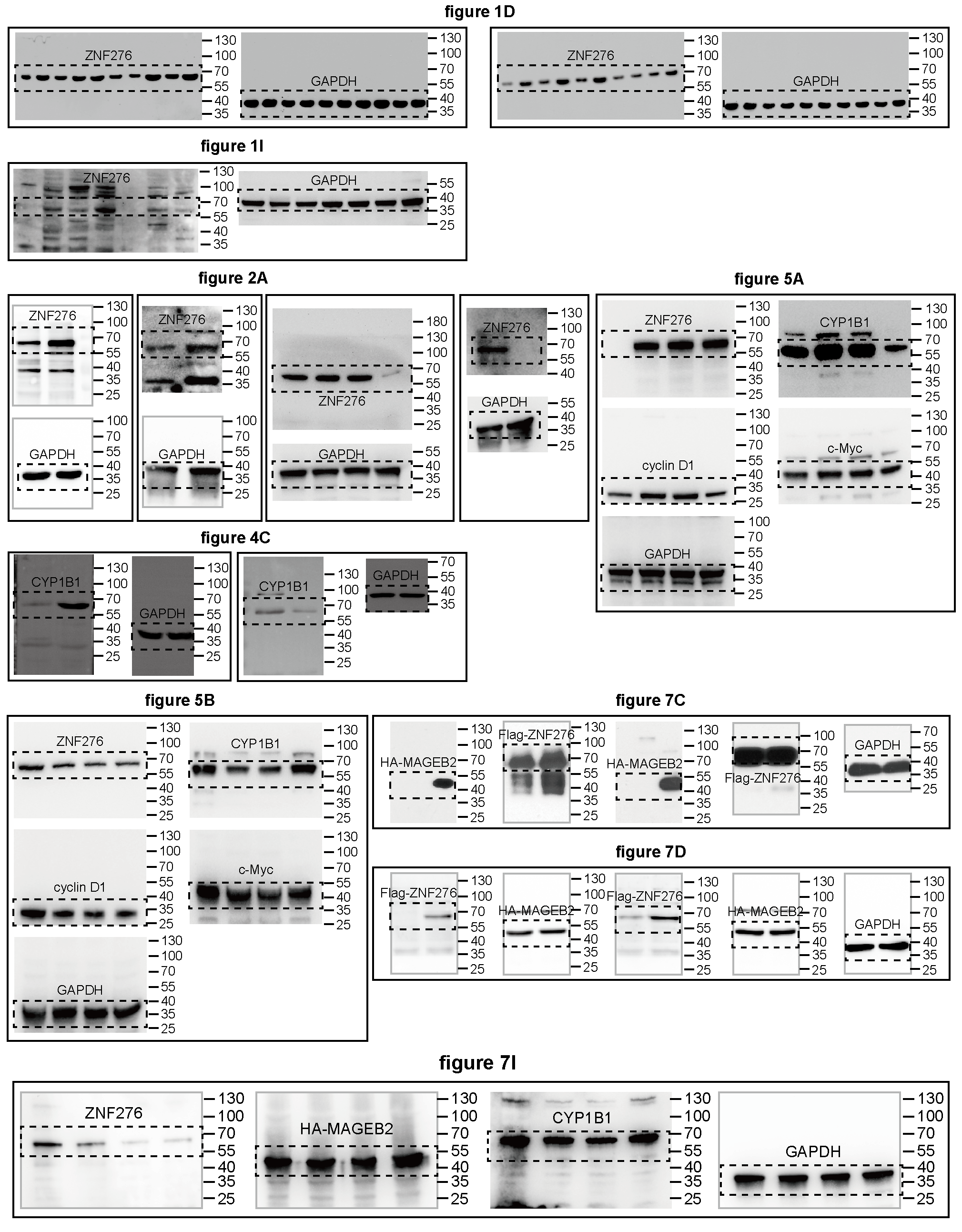

Supplement: Supplementary file 10 — Original Data File 1 [file 41419_2022_5223_MOESM10_ESM.tif]

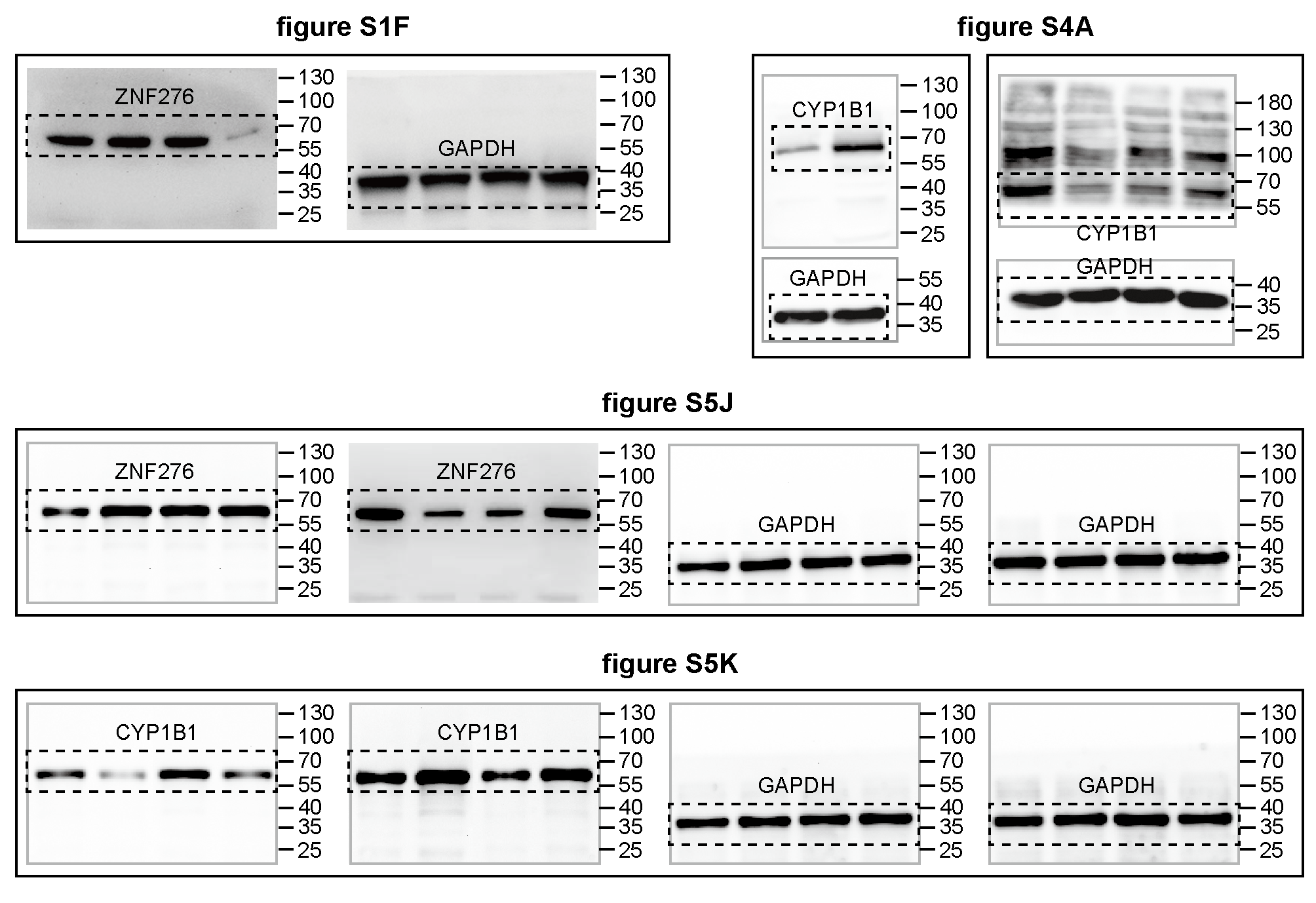

Supplement: Supplementary file 11 — Original Data File 2 [file 41419_2022_5223_MOESM11_ESM.tif]
