# Supplementary material for: ZNF276 promotes the malignant phenotype of breast carcinoma by activating the CYP1B1-mediated Wnt/β-catenin pathway
Source: Cell Death Dis. 2022 Sep 10;13(9):781. doi: 10.1038/s41419-022-05223-8 (PMC9463175; doi:10.1038/s41419-022-05223-8)
Supplement: Supplementary file 9 — Supplementary Figure Legends [file 41419_2022_5223_MOESM9_ESM.docx]

**Supplementary Figure Legends**

**Figure S1. Effects of ZNF276 on breast tumor growth in vitro and vivo. A.** Genome sequencing revealed deletion mutations in ZNF276 by sgRNA. **B, C.** Overexpression of ZNF276 in MDA-MB-231 (A) and SK-BR-3 (B) was detected by RT-qPCR. **D, E.** ZNF276 knockdown was also measured in MCF-7 (D) and MDA-MB-231 (E) by RT-qPCR. **F.** The efficiency of ZNF276 knockdown in MDA-MB-231 at protein levels was validated by western blot. **G, H.** CCK8 (G) and colony formation assays (H) measured the effect of ZNF276 deletion on cell proliferation in MDA-MB-231. **I.** ZNF276 overexpression affecting breast tumorigenesis in the mammary fat pad of nude mice.

**Figure S2. ZNF276 promoted the migration and invasion of breast carcinoma. A-C.** The motility of MDA-MB-231 cells overexpressed in ZNF276 was assessed by Transwell migration (A), invasion (B) and wound healing assays (C). **D-F.** Transwell migration (D), invasion (E) and wound healing assays (F) were also applied in SK-BR-3 cells overexpressed in ZNF276. **G-I.** The motility of MCF7 cells knockdown in ZNF276 was assessed by migration assays (G), invasion assays (H) and wound healing assays (I). **J-L.** The motility of UACC812 cells knockout in ZNF276 was assessed by migration assays (J), invasion assays (K) and wound healing assays (L). **M-O.** The motility of MDA-MB-231 cells knockdown in ZNF276 was assessed by migration assays (M), invasion assays (N) and wound healing assays (O). * *p* < 0.05, ** *p* < 0.01, *** *p* < 0.001.

**Figure S3. Identification and functional enrichment analysis of differentially expressed genes. A, B.** Volcano (left panels) and heat maps (right panels) were used to show the DEGs between ZNF276 overexpressing cells and the control cells. (n=3, biological replicates)**.** Significantly upregulated and downregulated genes were 2194 and 1979, respectively. **C-F.** Functional annotation and enrichment analysis of the ZNF276 transcriptome sequencing were used to demonstrate their potential biological functions, including Gene ontology (GO) analysis in biological process (C), cellular component (D), molecular function (E) and KEGG pathway analysis (F). The X axis shows the gene ratio. The left Y axis shows the top 10 positive biological function names. The darker the color represents the smaller the q value. Bubble size indicates DEG number.

**Figure S4. CUT&Tag analysis of ZNF276 target genes. A.** ChIPseeker analysis of the peak distribution. The pie chart shows that peaks are mainly distributed in distal intergenic, other intron and 1st intron. **B, C.** GO (B) and KEGG (C) analysis of the peak-associated genes were used to demonstrate their top 20 potential biological functions. **D.** The top 25 enriched DNA binding motifs identified by motif analysis software HOMER. **E.** Flow chart for screening of ZNF276 candidate target genes.

**Figure S5. Activation of the Wnt/β-catenin pathway by ZNF276. A-B.** ZNF276 lacking C2H2 domain inhibited the TOP Flash activity enhanced by the intact ZNF276 with wnt3a activation in MCF-7 (A) and HEK293T (B) cells.

**Figure S6. Verification of ZNF276 and CYP1B1 overexpression or knockdown and effect of CYP1B1 on BC cell proliferation, migration and invasion. A.** Western blotting analysis was used to confirm over-expression and knockdown of CYP1B1 in MDA-MB-231 or MCF-7 cells. **B-F.** Statistical analysis of CYP1B1 affecting breast cancer cell capacities of colony formation (B, D), migration and invasion (C, E), and wound healing (C, F). **G.** Representative pictures of CYP1B1 expression in 74 breast cancer tissues and 6 adjacent tissues from tissue chips. **H.** Detection of ZNF276 mRNA expression in various treatment groups in MDA-MB-231 and MCF-7 cells. **I.** Detection of CYP1B1 mRNA expression in various treatment groups in MDA-MB-231 and MCF-7 cells. **J.** Western blot analysis of ZNF276 expression in various treatment groups in MDA-MB-231 and MCF-7 cells. **K.** Western blot analysis of CYP1B1 expression in various treatment groups in MDA-MB-231 and MCF-7 cells. * *p* < 0.05, ** *p* < 0.01, *** *p* < 0.001.

**Figure S7. CYP1B1 is critical for ZNF276 mediated progression of BC. A-D.** Representative photograph of ZNF276 affecting breast cancer cell capacities of colony formation (A), invasion (B), migration (C) and wound healing (D) through regulation of CYP1B1. Bar = 100 µm or 200 µm. The statistical analysis of these experiments was shown in Figure 6H-O.
