# Supplementary material for: ZNF276 promotes the malignant phenotype of breast carcinoma by activating the CYP1B1-mediated Wnt/β-catenin pathway
Source: Cell Death Dis. 2022 Sep 10;13(9):781. doi: 10.1038/s41419-022-05223-8 (PMC9463175; doi:10.1038/s41419-022-05223-8)
Supplement: Supplementary file 12 — Table S1 [file 41419_2022_5223_MOESM12_ESM.docx]

**Tab S1. Clinical characteristics of paired breast cancer tissues and adjacent tissues.**

| **Group** | **Tissue type** | **Age** | **Gender** | **ER** | **PR** | **Her2** | **Pathogenic type** | **TNM stage** |
| --- | --- | --- | --- | --- | --- | --- | --- | --- |
| 1 | N/T | 35 | Female | - | - | + | IDC | T1 |
| 2 | N/T | 42 | Female | + | + | - | ILC | T2 |
| 3 | N/T | 40 | Female | + | + | - | ILC | T2 |
| 4 | N/T | 27 | Female | - | - | - | Metaplastic | M1 |
| 5 | N/T | 34 | Female | - | - | + | Metaplastic | M2 |
| 6 | N/T | 28 | Female | - | + | - | Mixed | N1 |
| 7 | N/T | 50 | Female | - | - | + | Mucinous | T2 |
| 8 | N/T | 51 | Female | - | + | - | ILC | T1 |
| 9 | N/T | 46 | Female | + | + | - | IDC | T2 |
| 10 | N/T | 47 | Female | + | - | - | Mixed | N1 |
