# Supplementary material for: ZNF276 promotes the malignant phenotype of breast carcinoma by activating the CYP1B1-mediated Wnt/β-catenin pathway
Source: Cell Death Dis. 2022 Sep 10;13(9):781. doi: 10.1038/s41419-022-05223-8 (PMC9463175; doi:10.1038/s41419-022-05223-8)
Supplement: Supplementary file 13 — Table S2 [file 41419_2022_5223_MOESM13_ESM.docx]

**Table S2. List of primers**

| **Si RNA sequences** | | | |
| --- | --- | --- | --- |
| Name | F’ | | R’ |
| Si-RNA-1 | GUGGAUCUGAUCACAUCCATT | | UGGAUGUGAUCAGAUCCACTT |
| Si-RNA-2 | GGACGAGUCCUUUGAGCCUTT | | AGGCUCAAAGGACUCGUCCTT |
| Si-RNA-3 | GAACCAAGAAUUCGGAAGATT | | UCUUCCGAAUUCUUGGUUCTT |
| **RT-qPCR primers** | | | |
| Name | F’ | | R’ |
| ADAM28 | AGGACATGCTGTGTGTGACC | | ACCCCAACCACAATGGAGAA |
| ATP6V1C1 | TTGCATGCGGCAACTTCAAA | | ACAAGCCAACCAAGACATCCA |
| BMP2 | GCCAAACACAAACAGCGGAA | | GGGAGCCACAATCCAGTCAT |
| BRSK1 | AGAAATATTTGTACCTGGTTCTGGA | | AGAACTTTCGGGCCTCCTTG |
| CDK6 | GGCTCTAACCTCAGTGGTCG | | GCAGCCAACACTCCAGAGAT |
| CYP1B1 | AGCCACAACGAAGAGTTCGG | | TGCTCGAATTCGCGGAAAAC |
| DACT2 | GCTCCTGTTCTGAGTCCACC | | CTCAAAGGGGAACAGGGCTT |
| GAPDH | GGTATGACAACGAATTTGGC | | GAGCACAGGGTACTTTATTG |
| GRAMD1B | GGTGCTGCTGGTCATCCTTA | | CTTGGAGCCTTAGACCCTGC |
| IL24 | CTGTGGACTTTAGCCAGCAGA | | TACCTGGCTCCAGAGAAGCA |
| KIF3C | CCCGCGAACTCAAGCTCAA | | GCACCAGTGGCTGGAACT |
| MX2 | GGCAAGGAGCTTCTGGGATT | | TTCTGGAGCATGCTAAGGGC |
| MCM10 | AAAATCCCCTGAGAAGTCTCCCC | | CTTGGTAGCGCAGGGACATC |
| NDRG2 | AATGGTGGTTCCATGCAGGC | | GCCGTATGGTGTCTCCACAG |
| SOCS3 | GTGGCCACTCTTCAGCATCT | | GTCCAGGAACTCCCGAATGG |
| TC2N | TTGAGTCGACGCTTTCCTCC | | TTTGCTTAGCCCAGGGGAAC |
| ZNF276 | GGCTGCAACAAGGTTTTCAT | | ATTTGGTGGACGAGAAGGTG |
| **Mutagenesis primers** | | | |
| Name | F’ | | R’ |
| ZNF276△Z | CGCGCCCTTGCAATGGGAGACTTCCAGCGGCTCCTGGGAG | | TCCCATTGCAAGGGCGCGGCCCG |
| ZNF276△C | GAGGAGCTTCCCACCATCCAGGACAAGGCCCTGCCCCTG | | GATGGTGGGAAGCTCCTCCCTC |
| ZNF276△Z+C | CTGGTAAGGACACTTGTATACAAGTGTCCTTACCAGGGC | | GCGGACCAGCACCCGTTCTTC |
| ZNF276△Z+Inter | CGGGCTCTCGCCATGGGTTACAAGTGTCCTTACCAGGGC | | CGGTTTCCTGTCTGCCAATTG |
| ZNF276△C+Inter | AAAGCTGGGTCGTTACTAGATGGTGGGAAGCTCCTCCCTC | | GAGCAGGGAATGGCATTGGTAAAATTG |
| pGL3-Basic-Region1 Deletion | GCTAGCCCGGGCTCGAGGGAGCTCAAAGTGCAGGGTTG | | ACCGGAATGCCAAGCTTATTGAGACTGGGGGTCGGTGAG |
| pGL3-Basic-Region2 Deletion | CGAGATTCGCAGCGCGAGAAGCCACCCCCGCCCAAG | | CTCGCGCTGCGAATCTCG |
| pGL3-Basic-Region3 Deletion | GCATGTAGGAAAGGGCGCTGTGCGTGCGCAGCCGAG | | GCGCCCTTTCCTACATGCTG |
| pGL3-Basic-Region4 Deletion | GCCTGGCAGGCGCGACTGGACAGCAGAAACTTCAACCCG | | CAGTCGCGCCTGCCAGGC |
| pGL3-Basic-Region5 Deletion | GGGGAGGCGACAGCAGAAGAGTGGCCTCTACGCGGG | | TTCTGCTGTCGCCTCCCC |
| pGL3-Basic-Region6 Deletion | CCACCTGGAGTGGCCTCTTAAACAACCAACCAGGGGC | | AGAGGCCACTCCAGGTGGC |
| pGL3-Basic-Region7 Deletion | AGAACCTAAGATAAAATTGATGAAGCGCGGTTACCG | | AATTTTATCTTAGGTTCTCGCAAAC |
| pGL3-Basic-Region8 Deletion | CCTCGATTGGAGGTGGCTGGATGGCGCGCTTTGACTC | | AGCCACCTCCAATCGAGGC |
| pGL3-Basic-Region9 Deletion | GCTAGCCCGGGCTCGAGGAAGGCATTTGGGCCTCTTATC | | ACCGGAATGCCAAGCTTGCTCTACCAGCAGGCTTTCATG |
| **CUT Tag index primers** | | | |
| i5 | TAGATCGC | | |
| i7 | TAAGGCGA | | |
| **ChIP-PCR primers** | | | |
| Name | F’ | R’ | |
| CYP1B1 | GATATGACTGGA | AGTGGCAGGAGG | |
