# Supplementary material for: ZNF276 promotes the malignant phenotype of breast carcinoma by activating the CYP1B1-mediated Wnt/β-catenin pathway
Source: Cell Death Dis. 2022 Sep 10;13(9):781. doi: 10.1038/s41419-022-05223-8 (PMC9463175; doi:10.1038/s41419-022-05223-8)
Supplement: Supplementary file 14 — Table S3 [file 41419_2022_5223_MOESM14_ESM.docx]

**Tab S3. Expression data of 379 genes in RNA sequencing and CUT-Tag analysis**

| **Genes** | **Log2FC**  **(RNA-seq)** | **Peak enrichment**  **(CUT-Tag)** | **Genes** | **Log2FC**  **(RNA-seq)** | **Peak enrichment**  **(CUT-Tag)** |
| --- | --- | --- | --- | --- | --- |
| ABCC5 | -1.01 | 4.84 | MKX | 1.62 | 4.39 |
| ABLIM1 | 1.88 | 2.88 | MLLT10 | 1.08 | 4.63 |
| ACOXL | -1.31 | 2.88 | MPDZ | 1.39 | 2.88 |
| ADAM28 | 1.41 | 4.46 | MPP5 | 1.24 | 3.70 |
| AFF3 | -6.88 | 6.14 | MRTFB | 1.14 | 4.39 |
| AGPAT4 | -1.04 | 5.26 | MSI2 | -5.10 | 2.88 |
| AHCYL2 | 1.04 | 2.88 | MSRB3 | -1.08 | 4.63 |
| AHR | 1.70 | 4.63 | MTSS1 | -1.99 | 4.63 |
| ALKBH8 | 1.69 | 6.03 | MX2 | 7.08 | 3.64 |
| AMN | -1.52 | 2.88 | MVB12B | 6.49 | 4.31 |
| ANGPTL2 | -1.40 | 3.57 | MYBL1 | 1.56 | 2.88 |
| ANKFN1 | -4.95 | 4.46 | MYO5C | -2.43 | 2.88 |
| AP2S1 | -1.14 | 3.64 | NADK2 | 1.56 | 2.88 |
| APBA2 | 2.56 | 5.17 | NBEA | 1.17 | 2.88 |
| APOL2 | 1.06 | 4.63 | NBN | 1.48 | 2.88 |
| APOO | 1.12 | 2.88 | NCK2 | 1.53 | 2.88 |
| ARHGAP32 | 1.98 | 7.81 | NCOA2 | 1.18 | 5.26 |
| ARHGEF1 | 1.35 | 4.46 | NDRG2 | 7.65 | 3.77 |
| ARID3A | -2.08 | 5.45 | NEDD4L | -1.50 | 5.17 |
| ARID4B | 1.25 | 4.31 | NEDD9 | 5.43 | 3.57 |
| ATE1 | 1.95 | 4.63 | NEO1 | 2.61 | 2.88 |
| ATM | 1.36 | 5.45 | NET1 | 1.21 | 3.77 |
| ATP2B1 | 1.30 | 6.14 | NFAT5 | 1.79 | 2.88 |
| ATP6V1C1 | 1.76 | 3.45 | NFATC1 | -1.48 | 2.88 |
| ATP8B2 | -3.41 | 2.88 | NGEF | -1.38 | 2.88 |
| ATRX | 1.23 | 2.88 | NIPAL2 | 1.67 | 2.88 |
| AXIN2 | -2.28 | 6.03 | NMNAT2 | -2.34 | 6.03 |
| BATF | 6.90 | 6.56 | NOMO2 | -1.21 | 2.88 |
| BAZ2B | 1.65 | 2.88 | NR3C1 | 1.56 | 2.88 |
| BBX | 1.01 | 3.77 | NSUN6 | 1.33 | 3.57 |
| BCAS3 | -1.22 | 3.45 | OLA1 | 1.20 | 3.64 |
| BCL11A | -6.38 | 5.00 | OLFML2B | -2.37 | 4.31 |
| BCR | -1.31 | 7.50 | ORC5 | 1.70 | 2.88 |
| BMP2 | 1.19 | 3.70 | OSBPL8 | 1.02 | 5.08 |
| BRSK1 | 1.29 | 4.39 | OTUB2 | 1.54 | 2.88 |
| C12orf75 | -1.19 | 3.57 | OXSR1 | 1.30 | 5.17 |
| C13orf46 | -1.09 | 4.55 | P2RX4 | -2.59 | 3.77 |
| C16orf45 | -1.50 | 3.64 | P4HA1 | 1.85 | 6.14 |
| C17orf97 | 1.46 | 5.17 | PAG1 | 2.08 | 3.70 |
| CABLES1 | 1.14 | 2.88 | PANK2 | 1.17 | 4.55 |
| CACHD1 | -3.47 | 2.88 | PAPPA2 | -4.06 | 4.31 |
| CACNA2D1 | 2.13 | 2.88 | PARD3B | 1.53 | 2.88 |
| CARMIL1 | 1.31 | 5.93 | PCNT | 1.42 | 4.31 |
| CASP7 | 1.18 | 5.36 | PDE12 | 1.32 | 2.88 |
| CCBE1 | -1.67 | 2.88 | PDE1C | -7.14 | 4.39 |
| CCDC85C | -1.07 | 6.56 | PDLIM5 | -2.07 | 3.70 |
| CDC42BPA | 1.20 | 2.88 | PDS5A | 1.39 | 6.25 |
| CDH4 | -2.96 | 2.88 | PEAK1 | 1.12 | 2.88 |
| CDK15 | 5.86 | 2.88 | PHF3 | 1.45 | 2.88 |
| CDK6 | 1.4 | 2.87 | PHGDH | 6.84 | 6.67 |
| CDKN2AIP | 1.51 | 2.88 | PHLPP1 | -1.26 | 2.88 |
| CDS1 | 1.64 | 2.88 | PLAUR | -1.59 | 4.39 |
| CELSR1 | -1.67 | 2.88 | PLB1 | 2.16 | 2.88 |
| CEP97 | 1.15 | 5.83 | PLCG2 | 3.37 | 6.56 |
| CHN1 | 1.24 | 3.64 | PLD5 | -6.38 | 8.06 |
| CHRM3 | -6.17 | 3.64 | PLEKHA3 | 1.35 | 3.51 |
| CHST15 | 1.42 | 4.55 | PNPLA7 | -2.53 | 5.36 |
| CHSY3 | -5.77 | 3.64 | POLE2 | 1.05 | 2.88 |
| CLASP1 | 1.92 | 8.33 | POLR3B | 1.71 | 5.00 |
| CLASP2 | 1.22 | 7.76 | PPFIBP2 | 1.04 | 8.87 |
| CMIP | -1.00 | 5.17 | PPP2R1B | 2.60 | 3.77 |
| CNTNAP3 | -3.97 | 3.70 | PRDM16 | 5.14 | 4.55 |
| COL12A1 | 2.64 | 6.03 | PRDM2 | 1.08 | 5.36 |
| COL13A1 | -2.37 | 2.88 | PRDM8 | -2.80 | 5.93 |
| COL4A2 | -2.35 | 2.88 | PRKAG2 | 1.40 | 3.57 |
| COL6A1 | 1.88 | 5.45 | PRKAR2A | 1.61 | 2.88 |
| COQ8A | -1.01 | 5.17 | PRKAR2B | -1.06 | 5.26 |
| CPE | 1.22 | 4.46 | PRKCA | 1.13 | 4.39 |
| CPLANE1 | 1.92 | 6.78 | PSG8 | 5.72 | 3.64 |
| CPNE4 | -5.65 | 6.90 | PTK7 | -3.24 | 4.24 |
| CPQ | -1.88 | 3.57 | PTPRG | 1.40 | 2.88 |
| CRADD | -1.31 | 4.39 | PVR | -1.58 | 3.57 |
| CREB5 | -3.23 | 4.46 | PXDN | -3.48 | 2.88 |
| CRISPLD2 | 1.87 | 2.88 | RAB32 | -1.05 | 2.88 |
| CRLF2 | 1.61 | 5.36 | RAB7B | 2.80 | 2.88 |
| CRLF3 | 1.01 | 3.70 | RAD18 | 1.69 | 5.83 |
| CRTAM | 5.16 | 2.88 | RALGAPA1 | 1.15 | 6.90 |
| CSF2RA | 1.81 | 5.74 | RALGAPA2 | 2.00 | 3.91 |
| CSNK1G1 | 1.04 | 3.70 | RANBP17 | 1.10 | 4.92 |
| CTIF | -1.19 | 2.88 | RAP1GAP | 1.95 | 3.51 |
| CXCL8 | -2.79 | 4.46 | RAP1GAP2 | -3.15 | 2.88 |
| CYB5R3 | -1.40 | 5.93 | RAPGEF6 | 1.92 | 3.51 |
| **CYP1B1** | **4.45** | **5.08** | RBM28 | 1.19 | 4.46 |
| DACT2 | -4.32 | 2.75 | RBMS1 | 1.03 | 3.70 |
| DEGS2 | -1.87 | 3.64 | RBPJ | 1.61 | 2.88 |
| DHRSX | 1.62 | 2.88 | RERE | -1.06 | 9.38 |
| DHX9 | 1.94 | 2.88 | RGS9 | -1.78 | 2.88 |
| DLG1 | 1.20 | 2.88 | RHOU | 1.52 | 3.70 |
| DNMT3A | -1.98 | 3.57 | RNLS | 3.13 | 2.88 |
| DOCK9 | 1.44 | 5.45 | ROBO1 | -2.74 | 3.51 |
| DST | 2.06 | 2.88 | RPH3AL | -1.66 | 3.57 |
| DUSP10 | 1.62 | 5.26 | RPL14 | 1.23 | 3.70 |
| DUSP22 | -1.01 | 3.45 | RRP36 | 1.29 | 5.36 |
| DYSF | -2.18 | 5.45 | RTTN | 1.10 | 4.63 |
| E2F3 | 1.33 | 4.31 | RUVBL1 | -1.19 | 2.88 |
| EDRF1 | 1.15 | 3.70 | S1PR5 | -3.06 | 3.77 |
| EIF4G3 | 1.13 | 5.26 | SAE1 | -1.14 | 6.90 |
| EIPR1 | -1.07 | 5.26 | SAYSD1 | 1.15 | 3.64 |
| EMB | -1.74 | 3.85 | SCCPDH | 1.29 | 5.83 |
| EPB41 | 1.05 | 3.64 | SDCCAG8 | -1.06 | 2.88 |
| EPC2 | 1.23 | 4.31 | SEMA6A | -1.08 | 4.92 |
| EPHA6 | -7.25 | 4.55 | SEPT6 | -1.83 | 2.88 |
| ESM1 | -10.21 | 2.88 | SERPINB7 | 1.81 | 2.88 |
| ESPN | 2.25 | 5.36 | SGMS1 | 1.27 | 3.77 |
| ETV5 | -1.88 | 4.48 | SH3KBP1 | 1.58 | 2.88 |
| FAM120B | 1.13 | 6.84 | SH3PXD2B | -1.72 | 4.10 |
| FAM126A | 1.21 | 2.88 | SHANK2 | -1.88 | 3.64 |
| FAM131C | 2.88 | -2.70 | SHC2 | 1.13 | 3.51 |
| FAM198A | 1.89 | 4.55 | SHH | 2.04 | 3.45 |
| FAM208A | 1.50 | 2.88 | SHQ1 | 1.12 | 3.57 |
| FAM227A | 1.40 | 2.88 | SIN3B | -1.56 | 4.63 |
| FAM84A | -8.10 | 6.03 | SLC13A3 | 3.53 | 4.39 |
| FARP1 | -1.06 | 2.88 | SLC16A9 | 2.96 | 3.77 |
| FBLN5 | -4.29 | 7.63 | SLC35F5 | 1.00 | 2.88 |
| FBXO9 | 2.13 | 2.88 | SLC40A1 | 3.29 | 7.38 |
| FCHSD1 | -1.17 | 6.14 | SLC41A2 | 1.42 | 6.03 |
| FGF1 | -2.48 | 5.45 | SLCO3A1 | 3.71 | 3.70 |
| FGF13 | -6.47 | 6.25 | SLTM | 1.10 | 6.03 |
| FGF5 | -5.71 | 6.14 | SMARCAD1 | 1.34 | 2.88 |
| FLVCR2 | 2.06 | 4.92 | SMC5 | 1.66 | 2.88 |
| FNIP2 | 1.28 | 6.67 | SMYD3 | -1.01 | 2.88 |
| FOXQ1 | -2.77 | 4.39 | SOCS3 | -2.01 | 2.79 |
| FRAS1 | -4.39 | 2.88 | SPART | 1.02 | 2.88 |
| FRMD3 | -2.38 | 3.57 | SREBF1 | -1.04 | 5.36 |
| FRMD4B | 1.49 | 2.88 | ST7 | 1.36 | 2.88 |
| FRYL | 1.27 | 3.64 | ST8SIA4 | -1.25 | 2.88 |
| FYN | -1.38 | 3.70 | STK38 | 1.52 | 4.67 |
| GABRE | 3.15 | 5.26 | STXBP5 | 1.10 | 6.78 |
| GAL | 3.28 | 2.88 | SUMF1 | -1.00 | 6.67 |
| GCNT2 | 1.31 | 5.00 | SVIL | 1.46 | 6.14 |
| GDPD5 | -1.30 | 4.63 | SYNE2 | 1.88 | 7.63 |
| GRAMD1B | 4.81 | 2.88 | SYNE3 | -2.52 | 5.74 |
| GRIK4 | 2.71 | 3.51 | TAB3 | 1.52 | 2.88 |
| GRK5 | -1.11 | 4.46 | TAF2 | 1.54 | 3.51 |
| GTPBP6 | 1.20 | 2.88 | TC2N | -6.05 | 3.62 |
| HAND1 | 3.20 | 4.46 | THOC2 | 1.29 | 2.88 |
| HEATR1 | 1.10 | 3.51 | TIGAR | 1.08 | 5.17 |
| HECTD1 | 1.33 | 3.64 | TKFC | -1.96 | 3.77 |
| HERC1 | 1.05 | 2.88 | TLE6 | -2.19 | 3.77 |
| HIF1AN | -1.14 | 4.39 | TMEM102 | -1.04 | 5.17 |
| HIST1H4K | 2.23 | 2.88 | TMEM154 | -2.26 | 3.57 |
| HLX | -1.68 | 4.39 | TMEM229B | -1.58 | 4.39 |
| HMGN5 | -2.93 | 6.14 | TMEM241 | -1.51 | 4.55 |
| HSP90AA1 | 1.17 | 6.78 | TMEM44 | -1.51 | 2.88 |
| HSPB8 | 3.25 | 6.78 | TMPRSS2 | 7.93 | 6.14 |
| ICE1 | 1.35 | 4.39 | TOGARAM2 | 2.00 | 7.26 |
| IDE | 1.11 | 2.88 | TOX2 | -1.28 | 5.26 |
| IFIH1 | 1.58 | 5.36 | TPD52L1 | -1.70 | 5.45 |
| IGFBP7 | -1.19 | 3.77 | TPK1 | 1.04 | 3.64 |
| IL1R1 | 3.11 | 5.26 | TRAPPC2 | 2.30 | 6.14 |
| IL24 | 2.2 | 2.88 | TRAPPC9 | -1.55 | 5.00 |
| INHBA | -2.32 | 4.63 | TRIM29 | 6.51 | 7.35 |
| IRF6 | 2.22 | 5.36 | TRIM33 | 1.34 | 4.63 |
| ITGA3 | -2.09 | 4.46 | TRIML2 | -4.92 | 3.70 |
| JMJD1C | 1.25 | 2.88 | TTC7B | -1.12 | 6.35 |
| KAT2B | 1.63 | 3.70 | TTLL5 | 1.10 | 6.25 |
| KAT6B | 1.31 | 5.26 | TUBGCP5 | 1.33 | 2.88 |
| KAZN | 4.22 | 5.08 | TULP4 | 1.02 | 4.46 |
| KCP | 2.57 | 2.88 | TYW1 | 1.77 | 3.70 |
| KIAA0753 | 1.65 | 2.88 | UBE4A | 1.90 | 3.64 |
| KIAA1109 | 1.30 | 3.77 | UBXN8 | 1.60 | 2.88 |
| KIAA1211 | -3.15 | 2.88 | USP24 | 1.30 | 3.70 |
| KIAA1522 | 1.17 | 5.45 | USP37 | 2.16 | 4.17 |
| KIF13A | 2.27 | 5.26 | UST | -3.33 | 5.45 |
| KIF26B | 2.75 | 4.84 | UTRN | 1.06 | 3.64 |
| KIF3C | -2.33 | 4.36 | VPS13C | 3.28 | 5.36 |
| KLHL29 | -1.25 | 6.06 | VPS13D | 1.52 | 4.55 |
| KMT2C | 1.35 | 5.36 | VPS36 | 1.31 | 5.45 |
| KSR1 | -1.12 | 5.36 | WASF3 | 2.41 | 2.88 |
| LAMC2 | -1.86 | 4.39 | WDFY3 | 2.02 | 5.36 |
| LANCL2 | 1.38 | 3.64 | WHRN | -1.38 | 5.00 |
| LBH | 2.83 | 3.57 | WSB1 | 1.56 | 2.88 |
| LIFR | 2.60 | 3.64 | XXYLT1 | -1.92 | 5.65 |
| LIMCH1 | -1.10 | 3.77 | ZBTB38 | 1.07 | 4.55 |
| LRRFIP2 | 1.34 | 5.65 | ZBTB40 | 1.02 | 2.88 |
| LRRIQ3 | -2.28 | 3.70 | ZBTB7C | 9.27 | 5.93 |
| LRTOMT | 1.14 | 6.14 | ZC3H12C | 1.11 | 4.46 |
| LTBP1 | -2.23 | 3.70 | ZC3HAV1L | 1.40 | 3.64 |
| LYRM9 | 1.13 | 11.97 | ZDHHC13 | 1.30 | 3.70 |
| MAP2K3 | -1.37 | 3.45 | ZFR | 1.89 | 7.26 |
| MAP3K2 | 1.97 | 6.67 | ZIC2 | -1.26 | 2.88 |
| MARK1 | -1.11 | 2.88 | ZNF180 | 1.01 | 3.70 |
| MAST4 | -1.20 | 3.70 | ZNF248 | 1.79 | 2.88 |
| MCM10 | 2.72 | 3.45 | ZNF326 | 1.68 | 2.88 |
| MEGF9 | 2.14 | 2.88 | ZNF354C | 2.21 | 6.14 |
| METTL8 | 1.43 | 2.88 | ZNF407 | 1.09 | 4.55 |
| MFNG | 1.92 | 2.88 | ZNF568 | 1.01 | 2.88 |
| MICALL2 | 1.37 | 4.46 | ZNF776 | 1.21 | 5.45 |
| MID1 | 1.02 | 3.57 | ZNF91 | 1.15 | 2.88 |
| MINDY4 | 1.01 | 4.46 |  |  |  |
